# Glioblastoma CUSA Fluid Protein Profiling: A Comparative Investigation of the Core and Peripheral Tumor Zones

**DOI:** 10.3390/cancers13010030

**Published:** 2020-12-23

**Authors:** Giuseppe La Rocca, Giorgia Antonia Simboli, Federica Vincenzoni, Diana Valeria Rossetti, Andrea Urbani, Tamara Ius, Giuseppe Maria Della Pepa, Alessandro Olivi, Giovanni Sabatino, Claudia Desiderio

**Affiliations:** 1 Department of Neurosurgery, Mater Olbia Hospital, 07026 Olbia, Italy; giuseppe.larocca@materolbia.com; 2 Institute of Neurosurgery, Fondazione Policlinico Universitario A. Gemelli IRCCS, Catholic University, 00168 Rome, Italy; giorgia.simboli573@gmail.com (G.A.S.); giuseppemaria.dellapepa@policlinicogemelli.it (G.M.D.P.); alessandro.olivi@policlinicogemelli.it (A.O.); 3 Dipartimento di Scienze Biotecnologiche di Base, Cliniche Intensivologiche e Perioperatorie, Università Cattolica del Sacro Cuore, 00168 Roma, Italy; federica.vincenzoni@unicatt.it (F.V.); dianavaleria.rossetti@unicatt.it (D.V.R.); andrea.urbani@unicatt.it (A.U.); 4 Fondazione Policlinico Universitario A. Gemelli IRCCS, 00168 Roma, Italy; 5 Neurosurgery Unit, Department of Neurosciences, University Hospital of Udine, 33100 Udine, Italy; tamara.ius@asufc.sanita.fvg.it; 6 Istituto di Scienze e Tecnologie Chimiche “Giulio Natta”, Consiglio Nazionale delle Ricerche, 00168 Roma, Italy

**Keywords:** brain tumor, glioblastoma multiforme, CUSA fluid, proteomics

## Abstract

**Simple Summary:**

The biological processes responsible for the high infiltration and recurrence rate of glioblastoma multiforme, the most frequent and aggressive primary brain tumor (GBM), are still under investigation. By the original analysis of cavitating ultrasound aspirator fluid as the biological specimen, the present study aimed to preliminarily explore and compare the protein profiles of the tumor core and tumor periphery, as defined by 5-aminolevulinic acid fluorescence, in newly diagnosed and recurrent glioblastoma sampled pools. The results showed distinguished protein elements in the different tumor and peritumoral zones, as well as in the two tumor states (newly diagnosed vs recurrent), and suggested the presence of pathological aspects in the fluorescent negative periphery, possibly contributing to the comprehension of the molecular mechanisms underlying this tumor’s onset and development, opening to potential clinical applications.

**Abstract:**

The present investigation aimed to characterize the protein profile of cavitating ultrasound aspirator fluid of newly diagnosed and recurrent glioblastoma comparing diverse zones of collection, i.e., tumor core and tumor periphery, with the aid of 5-aminolevulinic acid fluorescence. The samples were pooled and analyzed in triplicate by LC-MS following the shotgun proteomic approach. The identified proteins were then grouped to disclose elements exclusive and common to the tumor state or tumor zones and submitted to gene ontology classification and pathway overrepresentation analysis. The proteins common to the distinct zones were further investigated by relative quantitation, following a label free approach, to disclose possible differences of expression. Nine proteins, i.e., tubulin 2B chain, CD59, far upstream element-binding, CD44, histone H1.4, caldesmon, osteopontin, tropomyosin chain and metallothionein-2, marked the core of newly diagnosed glioblastoma with respect to tumor periphery. Considering the tumor zone, including the core and the fluorescence positive periphery, the serine glycine biosynthesis, pentose phosphate, 5-hydroxytryptamine degredation, de novo purine biosynthesis and huntington disease pathways resulted statistically significantly overrepresented with respect to the human genome of reference. The fluorescence negative zone shared several protein elements with the tumor zone, possibly indicating the presence of pathological aspects of glioblastoma rather than of normal brain parenchyma. On the other hand, its exclusive protein elements were considered to represent the healthy zone and, accordingly, exhibiting no pathways overrepresentation. On the contrary to newly diagnosed glioblastoma, pathway overrepresentation was recognized only in the healthy zone of recurrent glioblastoma. The TGFβ signaling pathway, exclusively classified in the fluorescence negative periphery in newly diagnosed glioblastoma, was instead the exclusive pathway classified in the tumor core of recurrent glioblastoma. These results, preliminary obtained on sample pools, demonstrated the potential of cavitron ultrasonic surgical aspirate fluid for proteomic profiling of glioblastoma able to distinguish molecular features specific of the diverse tumor zones and tumor states, possibly contributing to the understanding of the highly infiltrative capability and recurrent rate of this aggressive brain tumor and opening to potential clinical applications to be further investigated.

## 1. Introduction

Glioblastoma multiforme (GBM) is the most frequent and aggressive primary brain tumor. Despite the extensive surgical resection and radio-chemotherapy, it tends to recur with poor prognosis for the patient. The understanding of the biological processes that lead to the onset and recurrence of this disease through its molecular characterization, could allow the discovery of potential diagnostic and prognostic biomarkers and/or targets for biomolecular therapies, still unavailable for GBM. One of the possible analytical approaches to this purpose is proteomics. Proteomics aims at the identification of proteins expressed by a cell, a tissue or a biological fluid, revealing to be an essential tool, together with genomics, for the definition of the molecular features of a biological matrix. The protein phenotype of a cell does not strictly correlate with its genome expression profile, in fact, differently from the genome, the proteome changes. Protein alterations or post-translational modifications (PTMs) can occur under diverse environmental conditions and pathological states, or following epigenetic modulations, modifying their structure, biological function, and interactions. As a matter of fact, the processes involved in cellular transformation are often not directly correlated to the gene expression products but rather to their post-translational modified forms. 

Proteomics has already given consistent results in GBM research, both in evaluation of resistance to chemotherapy [1] and in the identification of potential biomarkers [2]. Recent reviews summarize the state of art of proteomic advances in GBM research [3,4,5] and the results from the characterization of extracellular vesicles (EVs) in its microenvironment [6,7]. However, to the best of our knowledge, no proteomics investigations on cavitating ultrasound aspirator (CUSA) fluid have been reported in GBM with the exception of the characterization of EVs [8,9,10]. In these papers the proteomic profile of EVs isolated in the aspirate fluid was investigated as a novel source of potential biomarkers.

A cavitating ultrasound aspirator (CUSA) allows tumor removal through ultrasonic tissue fragmentation under irrigation [11]. The fluid collected in the CUSA canister is composed of the saline solution used for irrigation, tumor tissue fragments and blood. This fluid is normally discarded while it has been conversely demonstrated to be an abundant and viable source of biological material, including dissociated tumor cells and fragments of tissue, useable for brain cancer research and overcoming the often poor availability of quantitative and good quality solid tumor material [12]. 

Apart from studies on CUSA EVs and on its derived cellular and solid tissue material, the proteomic profile of the soluble liquid fraction of CUSA fluid has never been investigated as far as we know in GBM or other brain tumors. 

In the present work we originally investigate CUSA fluid, after removal of cells and solid material, as an innovative biological matrix for a comparative proteomic study of GBM from diverse zones of resection, i.e., tumor core and peritumoral zones, by liquid chromatography-mass spectrometry (LC-MS) analysis in shot-gun approach. The use of CUSA fluid, representing the soluble fraction of the ultrasonic dissected tissue homogenates, could have interesting applications in the discovery of biomarkers by simplified procedures of sample pretreatment and direct application in the clinic, such as in intra-operative mass spectrometry tumor diagnosis. 

Many proteomic studies have been carried out on solid tumor tissue, however, one of the limitations of these studies is the overall poor availability of control samples of healthy tissue or the difficulty to differentiate tumor tissue from healthy peripheral zone. The present investigation, preliminarily performed on pooled samples of newly diagnosed (ND) and recurrent (R) GBMs, tried to overcome these main limitations by exploring the proteome of the CUSA fluid collected in three different zones, i.e., the core of the tumor, its periphery, and the surrounding macroscopically “healthy” as resulting from the aid of intraoperative 5-aminolevulinic acid (5-ALA) fluorescence, with the purpose to contribute either to the comprehension of the molecular mechanisms possibly implicated in the high infiltration and recurrence rate of this tumor, or to the discovery of new tools for tumor diagnosis.

## 2. Results

During the period of October 2018 and March 2019, seven patients were enrolled in the study and CUSA samples were collected during surgery. For each patient, three separate specimens were collected based on the diverse zones defined by 5-ALA fluorescence. Samples were then pooled into two main groups, newly diagnosed (ND) or recurrent (R) GBM, resulting in pooled samples in each group based on the different zones of collection, i.e., tumor core, 5-ALA positive and 5-ALA negative peritumoral zones, before LC-MS proteomic analysis. The analysis of pooled samples allowed to reduce the inter-individual variability and to provide a central proteomic profile for each group to be preliminarily investigated and compared, to provide a base for future individual sample investigations and validation. 

Before treatment, CUSA fluid was centrifuged to allow cells and solid material precipitation. Proteomic analysis was therefore applied to the resulting supernatant selectively containing the soluble fraction of CUSA proteome. This on one hand could have limited the characterization of less polar proteins and peptides, and of membrane proteins, but on the other hand, advantageously allowed proteomic characterization to be performed with reduced matrix interferences, suitable for the application of different analytical approaches and potential future applications such as intra-operative mass spectrometry tumor diagnosis and monitoring of the margins of resection. 

**Figure 1:**
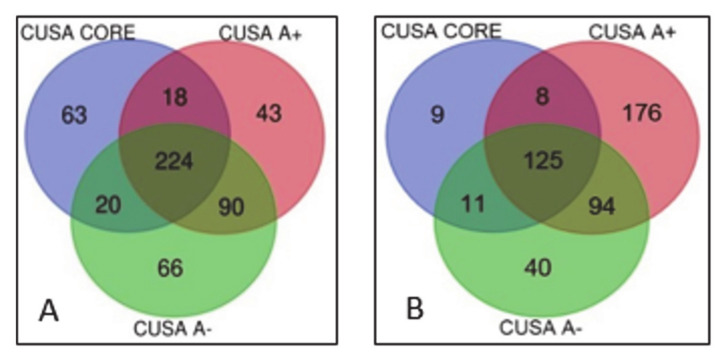
Identified proteins Venn diagram sample grouping in ND (panel A) and R (panel B) GBM pools.

The lists of the proteins and peptides identified in each of the three tumor zones pools of ND and R GBMs, as resulting from the multireport data elaboration of the analytical replicates by Proteome Discoverer software, are reported in Tables S1 and S2, respectively. These data have been filtered after elaboration, as specified in details in the materials and methods section, to ensure high confidence identification of proteins and peptides in the tumor zones and GBM states to be then analyzed and compared for functional interactions and gene ontology (GO) classification by bioinformatics tools. Figure 1 reports the Venn diagram grouping of the proteins identified in ND (panel A) and R (panel B) GBM groups in the relative diverse zone of collection. These groups of proteins were further submitted to bioinformatic data elaboration and comparison in order to disclose and discuss the differences between the GBM state and zones of sampling by studying their gene ontology (GO) classification, pathway categories, and overrepresentation analysis, either considering the common or the exclusive protein elements identified. The obtained results for each tumor group are described below in separate paragraphs and therefore compared and discussed. 

### 2.1. Newly Diagnosed (ND) GBM

As resulting from the ND GBM grouping of the proteins characterized in the diverse zones, out of the overall number of 463 unique elements identified with high confidence, 125 proteins were classified common to all tumor zones while 9, 176, and 40 elements were exclusive of CUSA CORE, CUSA A+, and CUSA A-, respectively (Figure 1A) (complete data are provided in Table S4). In addition, proteins shared by two different areas have been also observed and identified. Particularly CUSA CORE shared eight and 11 proteins with CUSA A+ and CUSA A-, respectively, while CUSA A+ and A- shared 94 proteins (Figure 1A). 

Focusing the attention on the ND GBMs Venn diagram (Figure 1A), different proteins relationships can be defined: proteins which may be considered definitely associated to the “tumor zone” and proteins associated to what may be considered the “healthy zone”, i.e., the proteins exclusively characterized in the 5-ALA negative periphery.

In order to define the tumor zone of the ND GBM, we considered the 9 proteins exclusive of CUSA CORE, the 176 proteins exclusive of CUSA A+ and the 8 proteins commonly identified in CUSA CORE and CUSA A+, for a total of 193 protein elements. Table S1 reports the Uniprot accession, the name and the zone of identification of the 193 proteins of the tumor zone. The proteins common between CUSA CORE and CUSA A- (11 elements) and between CUSA A+ and CUSA A- (94 elements) and the 125 proteins in common with all three zones (Figure 1A), were not included in the tumor zone as being in common with CUSA A-. 

The list of 193 protein elements characterizing the tumor zone was investigated for molecular pathways overrepresentation analysis by different bioinformatic tools providing complementary information, using the Homo sapiens dataset as reference. 

**Figure 2:**
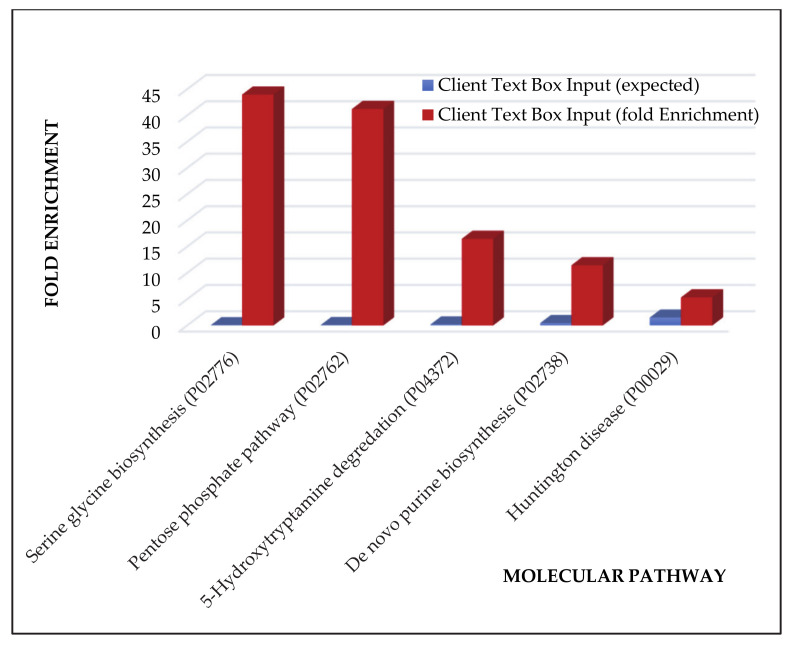
Pathway overrepresentation analysis of the protein elements characterizing the ND GBM tumor zone.

The gene-ontology overrepresentation analysis by PANTHER tool resulted in five pathways overrepresented (*p* value < 0.05), namely, in decreasing order of fold enrichment, serine glycine biosynthesis (P02776), pentose phosphate pathway (P02762), 5-hydroxytryptamine degredation (P04372), de novo purine biosynthesis (P02738), and huntington disease (P00029) (Figure 2).

The Reactome hierarchical pathway overrepresentation analysis in reference to Homo sapiens database found 168 identifiers over the 193 of the input list of the tumor zone and outlined the 25 most relevant pathways listed in Table 1, following a binomial test to calculate the result probability and by correcting the *p*-values for the multiple testing.

### 2.2. Recurrent (R) GBM

As it can be observed in the Venn diagram of R GBM (Figure 1B), the overall number of unique protein elements identified was higher than ND GBM, corresponding to 524 total. With respect to ND GBMs, a different distribution of the number of the protein elements exclusive of the different zones was also observed. Particularly, CUSA A- showed the highest number of exclusive protein elements (66), followed by CUSA CORE (63), and CUSA A+ (43) (Figure 1B) (complete data are available in Table S4). The most relevant differences with ND GBMs have been observed for CUSA CORE and CUSA A+, respectively showing a largely higher and lower number of exclusive proteins. 

These numbers allowed to interestingly compare their relative gene ontology classification and pathways overrepresentation analysis.

Differently from ND GBM pool, no statistically significant (*p* value >0.5) overrepresented pathways have been evidenced by PANTHER tool analysis in R GBM tumor zone. Differences between ND and R GBM pools were also recognized in the hierarchical pathways overrepresentation analysis by Reactome. Table 2 lists the 25 most relevant pathways overrepresented in R GBM tumor zone. 

## 3. Discussion

GBM is the most frequent and malignant primary brain tumor and, despite the multidisciplinary management, it highly relapses. The mechanisms underlying the high recurrence rate of GBM and therefore its aggressive behavior is still far from being fully understood. The new central nervous system (CNS) WHO 2016 classification divides CNS tumors on the basis of histological data integrated with molecular biology and genomic data that correlate with prognosis. The new nomenclature is formed by the histopathological name of the tumor followed by the genetic characteristics [13,14,15]. In this new classification, GBMs are divided in glioblastoma IDH-wildtype, 90% of cases, corresponding with what is defined as primary or de-novo glioblastoma, which predominates in patients over 55 years of age, glioblastoma IDH-mutant, about 10% of cases, which are closely related to the so-called secondary glioblastomas as are those with a history of prior lower grade diffuse gliomas preferentially occur in younger patient and glioblastoma NOS in tumors for which full IDH evaluation cannot be performed. In the present study the protein profile of the tumor core with its periphery, either positive or negative to 5-ALA fluorescence, was compared by pioneering investigating the soluble fraction of CUSA fluid, to discover potential differences and similarities. The main focus of the investigation was to discover distinct profiles for ND and R GBMs by analyzing CUSA fluid pools from IDH1 wild type GBMs, with special focus to the core region. In addition, within each GBM state, the presence of protein elements able to discriminate the tumor zone from the normal tissue was additionally investigated, together with the exploration of eventual pathological molecular profiles of the 5-ALA fluorescence negative tumor periphery, since the high infiltration capability of GBM. Although preliminary, this investigation originally performed on pooled samples, could contribute to the comprehension of the molecular mechanisms involved in GBM development and in its high infiltration capability in the surrounding zones, establishing new hints to be further investigated for potential in-surgery clinical applications. The obtained data are subsequently discussed by analyzing separately the results obtained from each GBM sample group and then compared. 

### 3.1. Newly Diagnosed GBM

#### 3.1.1. “Tumor Zone”

The gene ontology overrepresentation analysis of the ND GBMs “tumor zone” by PANTHER tool evidenced the overrepresentation of serine glycine biosynthesis, pentose phosphate pathway, 5-hydroxytryptamine degredation, de novo purine biosynthesis and huntington disease pathways. The role of the serine glycine biosynthesis pathway in cancer was recently described [16]. Although not specific for GBM, this pathway demonstrates to be involved in cancer growth and proliferation through its involvement in purine and pyrimidine nucleotides biosynthesis. The pentose phosphatase pathway (PPP) is also important, through its ribose-5-phosphate and NADPH products, for anabolic process and nucleic acid biosynthesis. This pathway undergoes a reciprocal metabolic switch with glycolysis, which is in association to cell proliferation and migration in glioma stem-like cells [17]. A recent study [18] demonstrated that self-renewing brain tumor initiating cells (BTICs) had elevated levels of metabolites of the de novo purine synthesis pathway, demonstrating the necessity of cell-promoting metabolism but also suggesting that BTICs may be sensitive to inhibition of the pathway.

The recognized overrepresentation of the 5-hydroxytryptamine degredation pathway could be consistent with literature data [19,20]. Studies in vitro on glioma cell lines interestingly showed enhanced cell proliferation following serotonin (5-hydroxytryptamine, 5HT) administration, exhibiting an invasion-promoting effect [19]. The administration of 5-HT receptor agonists resulted in a high inhibition of glioma stem cell proliferation and very recently serotonin receptors inhibitor drugs have been listed among the candidates for GBM therapies [20]. A review by Plun-Favreau et al. [21] discusses the various gene mutations that were found to be present and possibly link various neurodegenerative diseases, such as Huntington’s, with CNS tumors. Hence, it may be a possibility that GBM and Huntington’s disease have common pathways.

**Figure 3:**
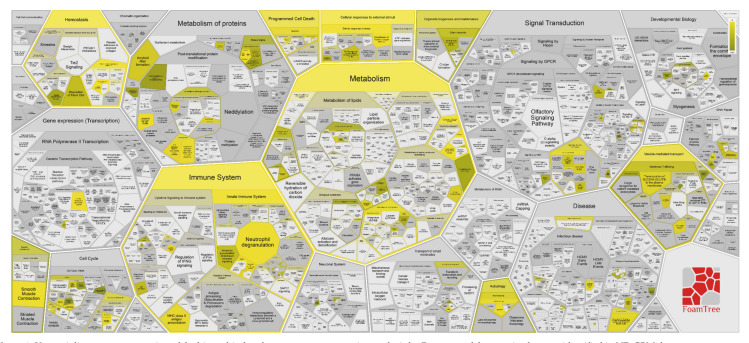
Voronoi diagram representation of the hierarchical pathways overrepresentation analysis by Reactome of the protein elements identified in ND GBM the tumor zone.

The Voronoi diagram visualization of the pathways overrepresentation analysis by Reactome shows that the 25 most relevant pathways of Table 1 lie inside the hierarchical signaling of metabolism, hemostasis, protein metabolism, gene expression, cellular responses to external stimuli, programmed cell death, cell cycle and immune system, muscle contraction, autophagy, transport of small molecules and vesicle-mediated transport main pathways (Figure 3). 

It is moreover relevant to focus on the nine proteins exclusively identified in ND GBM CUSA CORE, namely, tubulin 2B chain (Q9BVA1), CD59 glycoprotein (P13987), Far upstream element-binding (Q92945), CD44 antigen (P16070), histone H1.4 (P10412), caldesmon (Q05682), osteopontin (P10451), tropomyosin chain (P07951), and metallothionein-2 (P02795), and on the eight elements common to ND CUSA CORE and ND CUSA A+, and to outline their main molecular functions.

CD44 is a cell membrane glycoprotein involved in cell motility, proliferation, angiogenesis, and apoptosis [22]. CD44 was reported to take part in GBM playing a role in cell invasion and proliferation, tumor growth and inhibition of apoptosis [22]. CD44 was in fact hypothesized to be a marker of GBM cancer stem cells [22,23,24]. It was also shown to increase GBM resistance to radiation therapy [25]. A recent study demonstrated that a soluble CD44 protein secreted by the GBM cells is the link between GBM and neurodegenerative disorders, as it induces neuronal degeneration through the activation of tau pathology in the brain, which is known to be the underlying mechanism of some neurodegenerative disorders such as Alzheimer’s disease [26]. 

Osteopontin (OPN) is an intra- and extra-cellular glycophosphoprotein expressed in various cell types including macrophages, epithelial cells, smooth muscle cells, osteoblasts, cancer and immune cells, and plays a role in immune response [27]. In glioma perivascular niche, osteopontin was demonstrated to promote stem cell-like properties, as well as radiation resistance in adjacent tumor cells via the activation of CD44 signaling [24]. Furthermore, it is reported as one of the chemoattractants for GBM-associated microglia and macrophages [28]. This role is also reported by Wei et al. [27], discussing all the pathological roles of osteopontin upregulation in GBM, which includes promotion of angiogenesis through modulation of COX-2 expression, myeloid-derived suppressor cell expansion through the activation of STAT3 pathway and suppression of antitumor immunity by promoting extramedullary myelopoiesis [27].

CD59 has been found to be overexpressed in tumor cells as well as in immune cells involved in the microenvironment of the tumor. It is not specific of GBM, however possible targeted therapies inhibiting CD59 are currently under investigation for other types of cancers [29].

Caldesmon is a cytoskeleton-associated protein. Selected missplicing of exons of the gene are exclusively found in glioma microvessels leading to the upregulation of the protein level with proportional downregulation of tight junction proteins and effects on the permeability of GBM microvasculature [30]. This is a possible physiopathological mechanism for the edema present in GBM.

Tubulin 2B chain is overexpressed and specific to gliomas, as both its RNA and protein expression is highly represented in GBM [31,32,33]. 

Metallothionein-2 (MT2A) belong to the family of metallothioneins (MTs), cysteine rich low molecular weight proteins. The MT genes were recently reported to exhibit increased expression levels according to astrocytoma tumor grade. They resulted highly expressed in high grade astrocytomas and associated with shorter patient survival [34]. A recent review paper outlined the role of metallothioneins in cancer, discussing their involvement in tumor growth and proliferation processes, immune surveillance escape and drug resistance and evidencing their potential targeting for cancer therapy [35].

**Figure 4:**
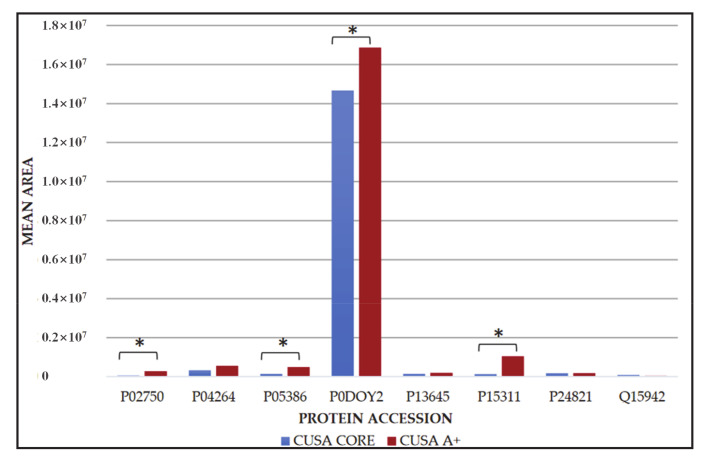
Bar chart representing the mean area with their corresponding standard deviations of leucine-rich alpha-2 glycoprotein (P02750), keratin, type II cytoskeletal 1 (P04264), 60s acidic ribosomal protein P1 (P05386), immunoglobulin lambda constant 2 (P0DOY2), keratin, type I cytoskeletal 10 (P13645), ezrin (P15311), tenascin (P24821) and zyxin (Q15942) proteins common to CUSA core and CUSA A+., The proteins with statistically significant differences of the mean area values in the two zones are marked. (*, *p*-value <0.05).

Relatively to the eight elements in common between CUSA CORE and CUSA A+, it was interesting to investigate potential statistically significant differences in their levels in the two zones by label-free quantitation and t-test evaluation. Figure 4 shows the relative quantitative data of the average protein area values of the three analytical replicates. Immunoglobulin lambda 2 constant (P0DOY2) protein, namely, is the much highly represented in both tumor zones compared to the other seven proteins. 

**Figure 5:**
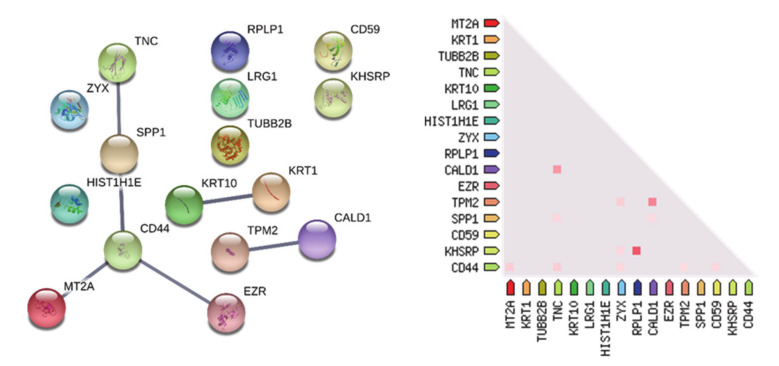
Protein-protein functional interaction network (medium confidence) of the 9 + 8 proteins discriminatory of the CORE tumor zone. The higher the thickness, the higher the confidence of the interaction. On the left the co-expression of the proteins in Homo sapiens is reported.

Out of the eight proteins, four of them showed statistically significant (*p*-value <0.05) different levels in the two tumor zones, namely: leucine-rich alpha-2 glycoprotein (P02750), 60s acidic ribosomal protein P1 (P05386), immunoglobulin lambda constant 2 (P0DOY2), and ezrin (P15311), all exhibiting higher levels in CUSA A+ compared to CUSA CORE. The 17 proteins including the nine exclusive of CUSA CORE and eight common to CUSA CORE and CUSA A+, were further investigated for protein-protein interaction networks functional enrichment analysis by STRING tool (Figure 5). 

The network revealed three main clusters of interaction, the most relevant involving tenascin-C(TNC), osteopontin (SPP1), CD44, metallothionein 2 (MET2A), and ezrin (EZR). It is noteworthy that the proteins involved in this cluster are mostly represented for the main part by exclusive proteins of the CUSA CORE (osteopontin, CD44, and MET2) and only one, ezrin, is in common, with statistically different level, with CUSA A+. In addition, inside the total group, co-expression in Homo sapiens is depicted (Figure 5). Various studies demonstrate that these proteins are involved in oncogenesis and/or GBM pathogenesis. Tenascin-C (TNC) has been demonstrated to have an angiogenic role in GBM [36]; ezrin to inhibit NF2 tumor-suppressor in GBM [37]. Zyxin has been demonstrated to play a role in cell migration and proliferation in various malignancies [38]. The role of zyxin in the invasive growth of GBM was recently demonstrated and its high expression was found to correlate with a worse prognosis [39].

**Figure 6:**
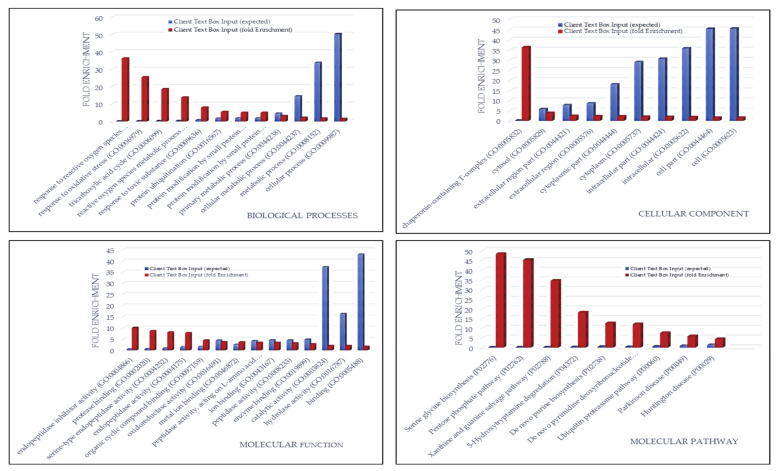
Bar charts of the gene ontology overrepresentation analyses carried out by PANTHER tool between the 176 exclusive proteins of ND GBM CUSA A+ and the Homo sapiens data of reference.

The 176 exclusive protein elements of CUSA A+ strongly influenced the molecular profile of the tumor zone because of the high number with respect to the exclusive proteins of CUSA CORE and their shared elements. Their gene ontology classification and overrepresentation of biological process, cellular component, molecular function and molecular pathways is shown in Figure 6 where the relative bar charts of the statistically significant (*p*-value of <0.05) results by PANTHER tool are shown. For each classification, the relative fold enrichment (in red) is reported with respect to Homo sapiens data of reference (in blue). In terms of biological process, cellular component, molecular function and protein class, either over- or under-representation were observed, both distinguishing, although in the opposite direction, the GBM pathological state from the normal condition. According to their high number of elements over the total identified in the tumor zone, the molecular pathway overrepresentation analysis of the proteins exclusive of CUSA A+ in Figure 6 showed very similar results to those obtained for the entire tumor zone, above discussed.

#### 3.1.2. “Healthy Zone”

The other elements of interest in ND GBM when looking at Figure 2 were the 40 proteins exclusively identified in what is considered the “healthy zone”, i.e., the CUSA A-. Interestingly, their overrepresentation analysis by PANTHER showed no pathways over-representation compared to human database of reference (*p*-value <0.05), suggesting a normal molecular profile and therefore a peritumoral 5-ALA negative area in general compatible with normal tissue. However, it is necessary to underline that these 40 proteins were only a small part out of the total 270 proteins that make up with confidence the CUSA A- proteomic profile, as the majority resulted in common with both tumor core and 5- ALA + tumor zones. These overlapping areas were therefore of great interest to investigate.

#### 3.1.3. Overlapping A- and CUSA CORE Zones

11 proteins were shared between CUSA CORE and CUSA A- zone (Table 3). Quantitative analysis was performed on this set of proteins in order to determine whether the relative mean areas were statistically different between one zone in comparison to the other by two-tailed t-test. The proteins in bold showed statistically different levels in the two zones, and specifically, the ones with higher amounts in CUSA A- are additionally marked.

It is important to address the various perspectives of interpretation regarding these data. First of all, the meaning behind the proteins being either statistically or non-statistically different in representation poses a question of how to interpret it, whether those statistically significant represent a distinction in pathology or non-pathology in one area compared to the other, or if the non-statistically significant depict equal representation of pathology or non-pathology. Unfortunately, it is not possible to be able to address these hypotheses with certainty, as it represents a blindspot in GBM literary investigations provided thus far. One way to possibly try and gain a general idea of which way of interpretation may be more correct, is to briefly investigate in further detail some of these proteins. Metallothionein-3 (MT3, uniprot accession P25713) is one of those proteins calculated to be statistically higher in CUSA A- zone, and the MT3 gene has been reported to be highly expressed in GBM compared to lower grade astrocytomas, and to correlate with lower prognosis of survival (*p*-value <0.05) [34], hence its characterization in CUSA A- would be consistent with a pathological index for GBM. Heterogeneous nuclear ribonucleoprotein D-like (hnRNPDL) (O14979) is, on the other hand, statistically higher in CUSA CORE compared to CUSA A-, and it was identified as over-expressed in GBM as reported in the pathology section of The Human Protein Atlas [31,32,40]. Investigating on the non-statistically different proteins, apolipoprotein CIII (P02656) and stathmin (P16949) are classified as cancer-related genes [31,41], the latter additionally identified as a poor prognostic factor for liver cancer [31,32,42]. The peptidyl-prolyl cis-trans isomerase NIMA-interacting 1, overexpressed in CUSA A- was also classified as a cancer related protein [31,41]. At this point, it is safe to induce that several of these proteins are indices of pathology irrespective of their quantitative significance in one zone or the other, and possibly indicating the presence in CUSA A- of pathological aspects of GBM rather than normal brain parenchyma. 

#### 3.1.4. Overlapping A- and A+ Zones

Ninety four (94) proteins are in common between CUSA A+ and CUSA A- zone (Figure 2). 58 out of them resulted quantitatively statistically different in mean area (*p*-value < 0.05). Generally, these proteins showed higher levels in CUSA A+ with the exception of 6 proteins that are statistically higher in CUSA A-, which are also listed in Table 4**.**

By investigating the six proteins statistically higher in CUSA A-, some of these have demonstrated to be a favorable prognostic marker in GBM such as Glutamate dehydrogenase 1 [31,32,43], while others, when over-expressed, have been associated with unfavorable prognosis in GBM such as Proliferating cell nuclear antigen [44] and synapsin-1 [31,32,45]. Synapsin-1, synaptosomal-associated protein 25 and β-synuclein are neurophysiological proteins [46,47,48] but, according to Human Protein Atlas Pathology, their overexpression is associated with glioma [31,32,45,49]. Hence, there is a statistically higher presence of these proteins in CUSA A- compared to CUSA A+ zones, but whether this quantity is higher than a normal brain parenchyma is not possible to deduce, since the lack of normal brain parenchyma data of control. On a similar note, Glutamate dehydrogenase 1, when highly expressed, has been a favorable prognostic factor [31,32,43], while Proliferating cell nuclear antigen, playing a crucial role in DNA replication and cell cycle regulation [50,51], is correlated with increasing tumor grade and decreasing patient survival rates when overexpressed [44]. 

The sharing of protein elements between the healthy zone and the tumor zone makes difficult to establish the A- zone as cancer free. It is furthermore noteworthy to underline that 5 out of the 40 exclusive proteins characterized in CUSA A- are enclosed in the Protein Atlas list of cancer related proteins, namely the macrophage migration inhibitory factor (P14174), protein S100-A8 (P05109), complement component C6 (P13671), calpastatin (P20810) and Tropomyosin alpha-1 chain (P09493). Possibly considering their exclusion, the 40 exclusive elements of the A- zone could disclose potential biomarkers to distinguish normal from pathological tissue, however, it seems clear that other parameters should be taken into consideration and applied in a panel, such as the peak area ratio of the proteins in common with the tumor periphery, i.e., the A+ zone, significantly differently expressed in the two zones. 

#### 3.1.5. ND GBM Gene Ontology Classification

**Figure 7:**
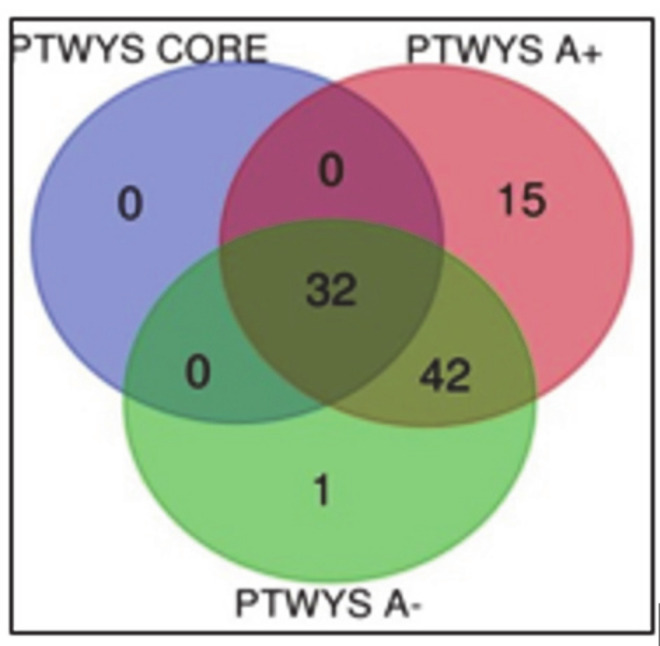
Venn diagram of molecular pathways from the three complete zones.

Apart from pathways overrepresentation analysis, the gene ontology classification of the molecular pathways was very different in number for each complete zone, namely, 32 in CUSA CORE, 89 in CUSA A+ and 75 in CUSA A-. Their Venn diagram elaboration found an overall number of 90 unique elements classified in the diverse zones under investigation (Figure 7, data in Table S5).

The 32 molecular pathways classified considering the CUSA CORE proteins, were all shared by CUSA A+ and CUSA A- pathways classification, hence, CUSA CORE did not exhibit exclusive pathways. Table 5 lists the molecular pathways exclusively classified in CUSA A+ and CUSA A-. 

It is not surprising that the pathways in bold in Table 5, i.e., the serine glycine biosynthesis and the 5-HT degradation pathways, which have been demonstrated involved in cell proliferation and migration, were included in the 5 over-represented molecular pathways of the “tumor zone”. 

What drew the attention was the single exclusive CUSA A- pathway, namely the transforming growth factor beta (TGFβ) signaling pathway, and its role in the pathogenesis of GBM. In fact, the GBM-associated microglial cells and macrophages (GAMMs), were reported to show not only classical macrophage activation aspects, but also typical hints of an alternative macrophage activation, the latter possibly involving inhibition of inflammation via production of TGFβ1, arginase 1 and interleukin 10, shaping the tumor micro-environment through vascular endothelial growth factor (VEGF) and matrix metalloproteases (MP). To date, it is well known that an abundance of GAMMs positively correlates with GBM invasiveness, immunosuppression, and the poor prognosis of the disease, calling for targeted immunotherapeutic strategies [28]. 

**Figure 8:**
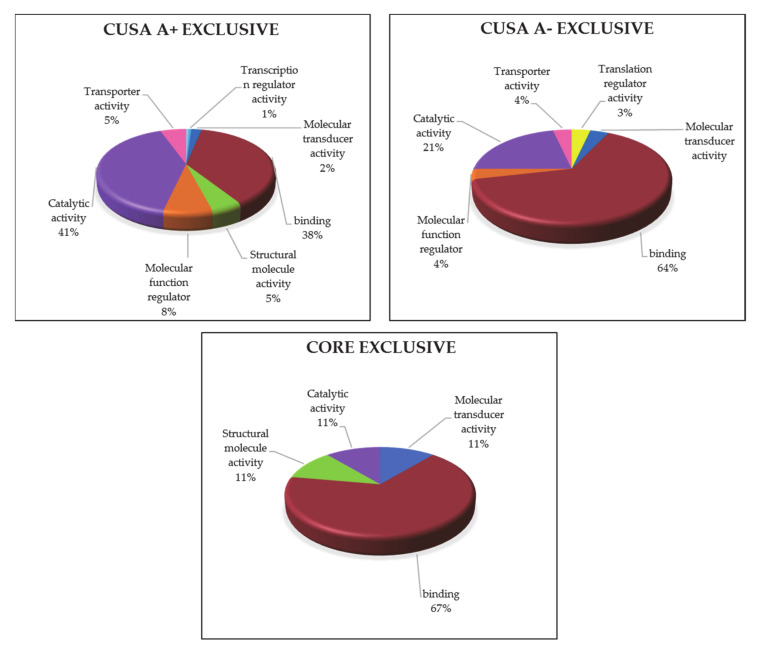
GO molecular function classification of the exclusive protein elements of each zone.

**Figure 9:**
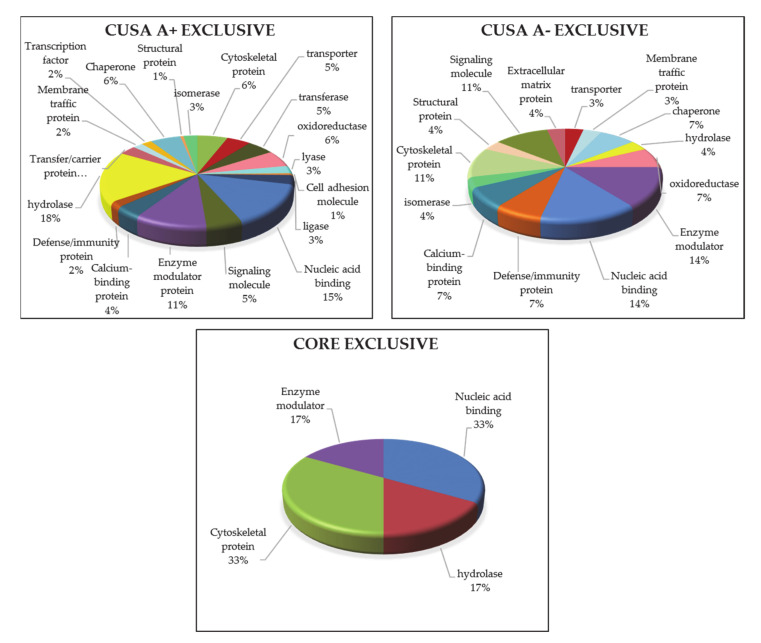
GO analysis of protein classes in each exclusive zone.

The gene ontology classification of molecular functions and protein class obtained for the protein elements exclusive of each zone was then performed and compared in Figure 8 and Figure 9, respectively. Looking at the molecular function (Figure 8) it is possible to state that the binding activity constitutes more than 60% of the functions for both CUSA CORE and CUSA A-, while CUSA A+ showed a higher number of proteins involved in catalytic activity that constitutes almost half of its functions. Apart from the classification of binding and catalytic activities, CUSA A- showed a molecular function classification more similar to CUSA A+, including molecular function regulator and transporter activity. One function that CUSA A- has exclusively is translation regulator activity, while one function shared among CUSA CORE and CUSA A+ is structural molecule activity.

The protein class categorization was very different among the three zones. CUSA CORE exclusive proteins consist in only four protein classes, also according to their low number, i.e., nine protein elements. One interesting finding is that in CUSA A- there is a higher representation of defense/immunity proteins, supporting the idea that the overall considered negative zone contains functions necessary to avoid pathological invasion. Another consideration is concerning hydrolase being present in CUSA A+ (18%), while only 4% present in CUSA A-. 

### 3.2. Recurrent GBM 

#### 3.2.1. “Tumor Zone”

Likewise and in comparison to ND GBM, the attention was at first focused on the protein elements characterizing the tumor zone of recurrent GBMs (R GBM), therefore considering the 124 total elements identified in the CUSA CORE, CUSA A+ as exclusive and in both zones, and on their molecular pathways overrepresentation analysis by PANTHER and Reactome tools. By comparing these results with those obtained for ND GBM, they were different, the first tool not evidencing pathways overrepresentation and the top 25 overrepresented pathways by Reactome resulting differently.

**Figure 10:**
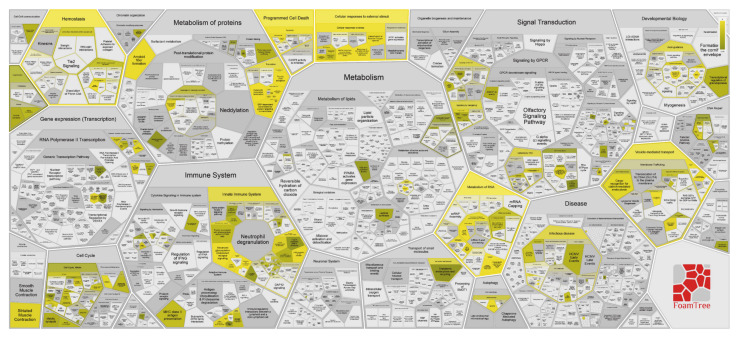
Voronoi diagram representation of the hierarchical pathways overrepresentation analysis by Reactome of the protein elements identified in the R GBM tumor zone.

Figure 10 shows the Voronoi diagram representation of the results obtained by the hierarchical Reactome pathways overrepresentation analysis of R GBM tumor zone. This representation evidences different overrepresented pathways inside the main hierarchical pathways of metabolism, immune system, protein metabolism. Furthermore, differently from ND GBMs, in R GBMs tumor zone the overrepresentation of pathways involved in RNA metabolism have been exclusively recognized. 

#### 3.2.2. “Healthy Zone”

The pathway overrepresentation analysis relative to the exclusive protein elements of CUSA A-, identifying the R GBM “healthy zone”, exhibited the statistically significant (*p* value < 0.05) overrepresentation of the xanthine and guanine salvage pathway, pentose phosphate pathway and TCA cycle, in decreasing order of fold enrichment. This result was different from ND GBM, where the “healthy zone” did not show pathways overrepresentation. 

PANTHER tool overrepresentation analysis of the “tumor” and the “healthy” zones therefore exhibited opposite results in ND and R GBM pools, particularly showing pathways overrepresentation in the tumor CORE and A+ zone in ND GBM and, on the contrary, only in the A- zone in R GBM. There was no over-representation of molecular pathways in R GBM “tumor zone”, drawing a more relevant attention of the R GBM tumor periphery and outlining the need of a deeper investigation on single specimen analysis. These results could be consistent with the high infiltration capability and the high diffusion and recurrence rate of GBM. 

#### 3.2.3. R GBM Gene Ontology Classification

Following the pathways overrepresentation analysis overview of the “tumor” and the “healthy” zones, the classification in molecular pathways within each complete group including the common proteins, have been furthermore evaluated and compared, as performed for ND GBMs.

**Figure 11:**
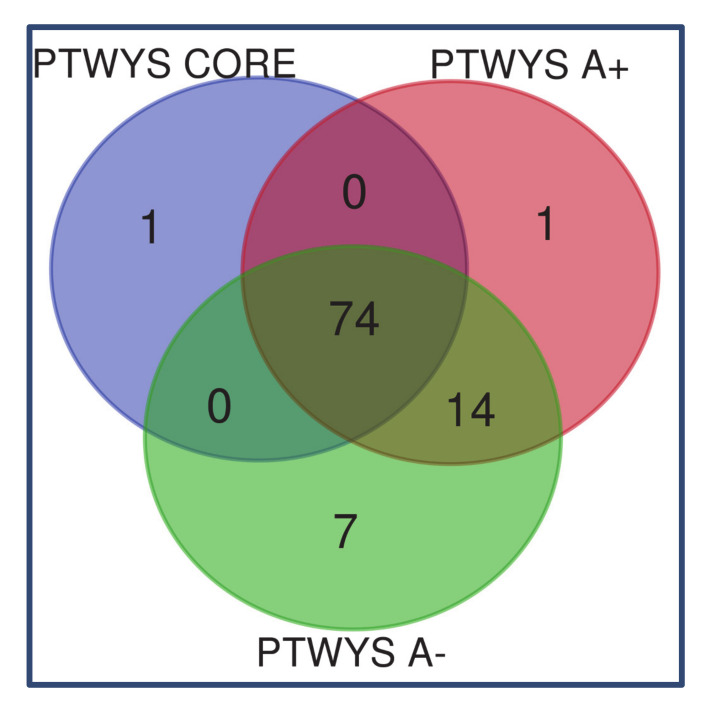
Venn diagram representation of pathways classified in the CORE, A+, and A- zones of R GBMs.

A total of 95 molecular pathways were classified for CUSA A-, 85 for CUSA A+, and 75 for CUSA Core (complete data in file Table S5) by PANTHER tool. The relative grouping by Venn diagram is reported in Figure 11 and found an overall number of 97 unique elements. The molecular pathways exclusively classified in each zone are reported in Table 6. 

It is very interesting to underline that the majority of pathways (*n* = 74) were commonly classified in all analyzed zones and that the CUSA CORE and CUSA A+ distinguished for only one exclusive pathway each, i.e., the TGFβ signaling pathway and the pathway of mannose metabolism, respectively. CUSA A- showed the highest number of exclusive pathways (*n* = 7). The most eye-catching information is the TGFβ signaling pathway as the unique exclusive molecular pathway classified in CUSA CORE, that, on the contrary, was the only exclusive molecular pathway of CUSA A- in ND GBM, thus again confirming an opposite result of ND and R GBM pools analysis.

From what has been discussed thus far, TGFβ signaling pathways is involved and dysregulated in GBM by inducing glioma invasion and migration, cell proliferation, angiogenesis and tumor-induced immunosuppression. Its classification in R GBM CORE and ND GBM A- zone, correlated to no pathway overrepresentation in these zones, is of controversial interpretation on the role of TGFβ signaling pathway in the progression and recurrence of GBM to be deeply investigated in future studies on individual samples.

The only exclusive pathway for CUSA A+ was the mannose metabolism. A crucial enzyme in mannose metabolism is mannose phosphate isomerase, which catalyzes an interconversion of fructose-6-phosphate and mannose-6-phosphate necessary to maintain levels of mannose in order to either enter the glycolytic pathway or form glycoproteins and polysaccharides, respectively. According to the Human Protein Atlas this enzyme is highly expressed in gliomas as well as is an unfavorable prognostic marker in head and neck cancers [31,32,52].

The seven pathways resulting exclusively in CUSA A- are interesting as are majorly involved in pathological progression. Two of the most relevant pathways are the endothelin signaling pathway and the PDGF signaling pathway. Endothelin signaling pathway leads to the production of nitric oxide (NO) and prostacyclins (PGI2) necessary for mitogenesis and differentiation primarily affecting endothelial and vascular smooth muscle cells, hence its presence is necessary for angiogenesis [53,54]. PDGF signaling pathway is actually very important as it recruits adaptor molecules via SH2 domains, leading to phosphorylation of other cellular proteins, hence plays a critical role in cellular proliferation and development [53,55], and has been implicated in GBM, thus many specific therapeutic target trials are currently under investigation [56]. Hence, it is again supposed that 5-ALA negative zone in R GBM seems to have pathological characteristics.

### 3.3. Newly Diagnosed versus Recurrent GBM

Following the separate discussion of the proteomic results obtained for ND and R GBMs, the relationships of the characterized proteins from each zone between the two GBM groups were additionally investigated. The main interest was drawn to the comparison of the full list of proteins characterizing the two core zones each including both the exclusive and the shared elements with the other analyzed zones and corresponding to 153 and 325 proteins in ND and R COREs, respectively. 

**Figure 12:**
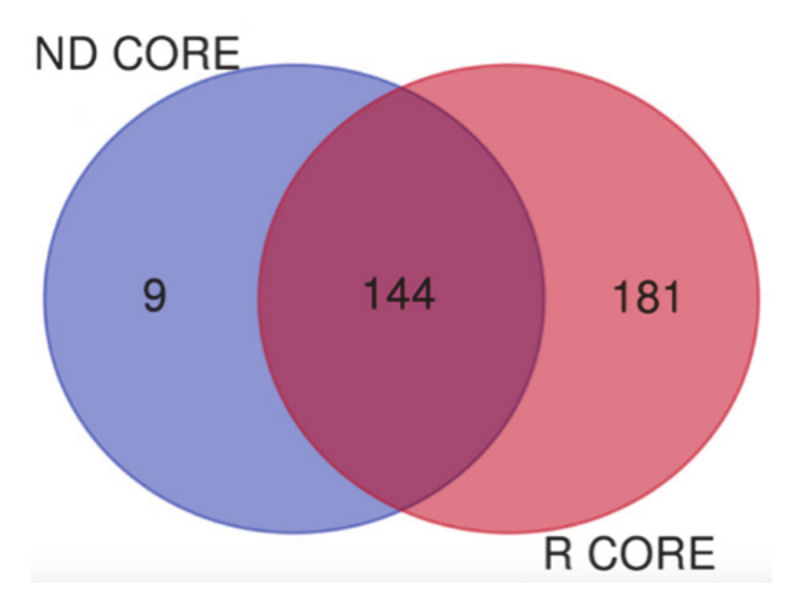
Venn diagram between the 153 ND Core proteins and 325 R Core proteins.

As depicted in the Venn diagram of Figure 12, the majority of the protein elements resulted in common between the ND Core and R Core, and nine proteins were exclusive of ND core, the same number of exclusive proteins that distinguished the CORE from the other zones inside the ND GBM group. 

Table 7 reports the Uniprot accession numbers and the names of the nine proteins distinguishing the ND GBM CORE from the R GBM CORE (column A), in comparison to the nine proteins differently distinguishing the ND GBM CORE from the ND other zones (column B). 

As can be seen in Table 7 the two lists share four proteins (in bold), which could be possibly capable to either distinguish ND CORE from the R CORE or ND CORE from its tumor periphery, namely ND A+ and ND A- zones. Differently, the other five proteins, that are instead exclusive only in their respective comparative scenarios, must be further investigated as potential specific markers distinguishing the cores of ND and R GBMs or the core of ND from the other ND zones. This finding could have a relevant diagnostic value, particularly if the data can be obtained from other biological samples of low invasiveness collection (i.e., blood or saliva) in evaluating an early relapse of GBM, for which to date MRI images are unable to fully distinguish an actual GBM relapse from pseudoprogression. In addition, from a surgical point of view, the so called “healthy” zone could be the limit of our surgery. It leads us to hypothesize that an intraoperative mass spectrometry, together with current tools (i.e., intraoperative MRI, contrast enhanced ultrasound, and neuronavigation) could drive surgery to a maximal safe resection. 

## 4. Materials and Methods

### 4.1. Patients Enrolment

Seven consecutive patients (median age 57.7 ± 12.7 years) with clinical and radiological data compatible with high grade glioma were included, under written consent, in the present study which is part of a study aiming to identify potential biomarkers of GBM. All the investigated GBMs were IDH1 wild type. Patient data are reported in Table 8. The study was approved by Ethical Committee of Catholic University of Rome with number 13891/18 ID 2015. According to our internal protocol, clinical and radiological data from every patient were collected. The CUSA fluid specimens were collected from newly diagnosed (ND, median age 64.5 ± 11.7 years) and recurrent (R, median age 48.7 ± 8.1 years) GBM patients (Table 8) and pooled accordingly to both GBM state and tumor zone of sampling as following specified. 

### 4.2. Chemicals

5-aminolevulinic acid (5-ALA) was from Medac (Wedel, Germany). Iodoacetamide (IAA), d,l-dithiothreitol (DTT), Ammonium bicarbonate (AMBIC), bovine serum albumin and acetone were from Sigma-Aldrich (St. Louis, MO, USA). Water, acetonitrile (ACN), formic acid (FA) were from Merck (Darmstadt, Germany). All organic solvents were of LC-MS grade. Trypsin enzyme (Gold MS Grade) was from Promega (Madison, WI, USA). 

### 4.3. Surgical Procedure and CUSA Aspirate Fluid Sampling

In our hospital we currently use the following set while doing brain surgery: 1) navigated contrast enhanced intraoperative ultrasound which, because of broken BBB, enhances the same region of 5 ALA+. 2) neuronavigation that allows to constant and better define the region in which we are performing surgery [57,58]. We are of course aware of the brainshift that can alter our images, but intraoperative reconstruction with ultrasound helps us to reduce the brainshift effect on our neuronavigation system. Following the standard protocol applied for patients with suspected GBM, 5-ALA in conventional dose of 20 mg/kg was administered approximately 6 h before the estimated time of having the tumor exposed [59,60,61]. After craniotomy and dural opening, the operating microscope (M530 OHX, Leica, Wetzlar, Germany) was used for tumor removal, switching from white light to fluorescence-enhanced vision just to gain the maximal safe resection. The samples were collected from the core of the tumor (CUSA CORE), the 5-ALA positive (CUSA A+) and the 5-ALA negative (CUSA A-) periphery. We used 5ALA, together with intraoperative contrast enhanced ultrasound and neuronavigation that matches the criteria of central part of the tumor (CORE), periphery with still uptake of 5ALA (5ALA+) and periphery without uptake of 5ALA but still suspicious for pathology or border territory (5ALA-). With the aid of neuronavigation and 5-ALA fluorescence, three areas of sampling were defined: the core of the tumor (CUSA CORE), the 5-ALA positive (CUSA A+) and the 5-ALA negative (CUSA A-) periphery [62]. During tumor resection, CUSA was configured with the following standard setting: 50% amplitude, 50% aspiration, and 3mL/min of irrigation with normal saline solution. CUSA (Integra: Tullamore, County Offaly, Ireland) was used for tissue resection from each of the three zones in a continuous manner for about 30 s. The fluid collected in the aspirator bag (Integra) was then extracted in a sterile manner and stored at −80 ^°^C until LC-MS proteomic analysis. A new bag was used for each zone of CUSA collection.

### 4.4. CUSA Fluid Sample Treatment

The samples were thawed at room temperature and subsequently centrifuged at 1200 rpm, 4 °C, for 5 min to allow the precipitation of cells and solid material. The resulting CUSA supernatant fluids were recovered and subsequently pooled based on tumor diagnosis, ND or R, and tumor zone of collection, i.e., core, 5-ALA positive, and 5-ALA negative peritumoral zones, by sampling identical volume aliquots. CUSA pools underwent UV-Visible (8453 UV-Vis Supplies, Agilent Technologies, Waldbronn, Germany) spectrophotometric analysis by Bradford Assay (Bio-Rad Laboratories, Hercules, CA, USA) to determine the total protein content using bovine serum albumin as standard of reference for calculation. 

A sample volume of each CUSA fluid pool corresponding to 50 µg of total protein content underwent the protocol treatment for shot-gun proteomic analysis. The sample was added 1:1 (**v/v**) of 200 mM DTT in AMBIC 100 mM and incubated at 100 °C for 5 min, and then at 50 °C for 15 min in thermomixer. The sample was following added 1:1 (*v/v*) of 200 mM IAA in 100 mM AMBIC and incubated at room temperature for 1h in the dark. Then 200 mM DTT in 100 mM AMBIC was added 1:1 (*v/v*) with respect to the IAA solution volume added in the step before. Protein digestion was allowed by addition of trypsin stock solution 1 µg/µL 1:50 (*w/w*) with respect to the total protein content of the sample volume treated. The sample was incubated for digestion at 37 °C for18 h. A volume of 1 µL of concentrated FA (100%) was added in order to stop protein digestion. The sample was then lyophilized, redissolved in 0.1% aqueous FA solution. The sample was analysed by LC-MS after dilution in order to inject 1 µg of total protein content into the chromatographic column. 

### 4.5. LC-MS Proteomic Analysis

LC-ESI-MS/MS shot-gun analyses were performed for each sample in triplicates on UltiMate 3000 RSLCnano System coupled to Orbitrap Elite MS detector with EASY-Spray nanoESI source (Thermo Fisher Scientific, Waltham, MA, USA) and Thermo Xcalibur 2.2 computer program (Thermo Fisher Scientific) for instrumental operation and data acquisition. EASY-Spray columns 15 cm in length × 50 µm of internal diameter (ID) were used, and PepMap C18 (2 µm particles, 100 Å pore size) (Thermo Fisher Scientific) was used for shot-gun analyses hyphenated with Acclaim PepMap100 nano-trap cartridge (C18, 5 μm, 100 Å, 300 μm i.d. × 5 mm) (Thermo Fisher Scientific) operating pre-separation peptide trapping and concentration. Chromatographic separations were performed at 40 °C in gradient elution using 0.1% FA as eluent A and an ACN/FA solution (99.9:0.1, *v/v*) as eluent B as following: i) 5% B (2 min), (ii) from 5% to 60% B (120 min), (iii) from 60% B to 99% (15 min), (iv) 99% B (10 min), (v) from 99% to 5% B (2 min), (vi) 5% B (13 min). The mobile phase flow rate was 0.3 μL/min. The injection volume was 5 μL. The Orbitrap Elite instrument was operating in positive ionization mode at a 60,000 full scan resolution in 350–2000 *m/z* acquisition range, performing MS/MS fragmentation by collision-induced dissociation (CID, 35% normalized collision energy) of the 20 most intense signals of each MS spectrum in Data-Dependent Scan (DDS) mode. The minimum signal was set to 500.0, the isolation width to 2 *m/z* and the default charge state to +2. MS/MS spectra acquisition was performed in the linear ion trap at normal scan rate.

### 4.6. Data Elaboration

LC-MS and MS/MS data were elaborated by Proteome Discoverer 1.4 software (version 1.4.1.14, Thermo Fisher Scientific), based on SEQUEST HT cluster as search engine against the Swiss-Prot Homo Sapiens proteome (UniProtKb, Swissprot, homo+sapiens released in March 2018). The following parameters were set: minimum precursor mass 350 Da; maximum precursor mass 10,000 Da; total intensity threshold 0.0; minimum peak count 1; Signal to Noise (S/N) threshold 1.5; mass tolerance 10 ppm; fragment mass tolerance 0.5 Da; use average precursor mass False; use average fragment mass False. Trypsin enzyme was set with a maximum of 2 missed cleavage sites. For data elaborations, the minimum and maximum peptide length was 6 and 144 residues, respectively. Dynamic methionine oxidation (+15.99 Da) and static carbamidomethylation of cysteine (+57.02 Da) were also set. Protein and peptide spectra matches were validated by the calculation of false discovery rate (FDR) using the Percolator node. The strict target FDR value was set at 0.01, while the relaxed value was set at 0.05. Protein identification results were further filtered for high peptide confidence identification; peptide rank 1; 2 peptides per protein; peptide length ≥ 9 amino acid residues, according to the Human Proteome Project Mass Spectrometry Data Interpretation Guidelines [63]. 

Sample data grouping analysis was performed by Venn diagram tool (http://bioinformatics.psb.ugent.be/webtools/Venn/). Gene Ontology (GO) classification, and pathways over-representation analysis of the identified proteins were performed by Protein ANalysis THrough Evolutionary Relationships (PANTHER, http://www.pantherdb.org) Classification System (version 11.0) [53] using Fisher’s Exact test type and correction of false discovery rate (FDR) and Reactome [64]. Protein-protein functional interaction networks have been investigated through STRING database [65]. In order to determine the quantification of protein presence in the different zones and hence to determine their statistically significant difference, the mean protein area values calculated by Proteome Discoverer software have been submitted to T-test statistical analysis, considering a *p*-value <0.05 as statistically significant. 

## 5. Conclusions

CUSA fluid is an innovative biological matrix that can be successfully applied for molecular characterization studies. The shotgun proteomic analysis of CUSA fluid, originally presented in this investigation, allowed to disclose distinguished molecular profiles associated either to the tumor state, i.e., newly diagnosed or recurrent glioblastoma, or to the diverse zones, namely the tumor core and the 5-ALA positive and negative tumor periphery. The share of protein elements and molecular pathways associated to glioblastoma between the healthy and the tumor zones could make possible the hypothesis of the presence of pathology in the fluorescence free detected zones, that it could be consistent with the high infiltration and recurrent rate of this aggressive brain cancer. Newly diagnosed and recurrent glioblastoma seemed to exhibit distinct molecular features of the CORE and A- zones. In light of this, CUSA fluid could be a novel source of potential biomarkers. Although preliminary, these results, overall obtained on CUSA pools, provided a first overview of the proteomic characterization of CUSA fluid and of the profiles associated to the diverse sample groups by reducing the inter-individual variability of the biological specimens. Further investigations are however necessary in order to confirm these data by screening individual specimens to allow an appropriate validation and to explore the application of CUSA fluid for clinical purposes. 

## Figures and Tables

**Table 1:** Result of the 25 most relevant Reactome pathways overrepresented in ND GBM tumor zone in *p*-value decreasing order.

Pathway Name	Entities Found	Entities Total	Entities Ratio	Entities *p*-Value	Entities FDR	Reactions Found	Reactions Total	Reactions Ratio
Innate Immune System	51	1187	0.103940455	5.27 × 10^−10^	5.64 × 10^−7^	131	662	0.052414885
Neutrophil degranulation	30	480	0.042031524	1.13 × 10^−9^	6.06 × 10^−7^	10	10	7.92 × 10^−4^
Platelet degranulation	14	128	0.011208406	6.47 × 10^−8^	2.30 × 10^−5^	6	11	8.71 × 10^−4^
Response to elevated platelet cytosolic Ca2+	14	133	0.011646235	1.03 × 10^−7^	2.74 × 10^−5^	6	14	0.001108472
Detoxification of Reactive Oxygen Species	8	39	0.003415061	4.89 × 10^−7^	1.05 × 10^−4^	11	32	0.00253365
Cellular responses to external stimuli	27	579	0.050700525	2.63 × 10^−6^	4.69 × 10^−4^	50	255	0.020190024
Cellular responses to stress	26	565	0.049474606	5.20 × 10^−6^	7.90 × 10^−4^	47	224	0.01773555
Immune System	68	2398	0.209982487	6.58 × 10^−6^	8.75 × 10^−4^	254	1598	0.126524149
Platelet activation, signaling and aggregation	16	265	0.023204904	1.66 × 10^−5^	0.001955007	9	115	0.009105305
Hemostasis	28	726	0.06357268	5.36 × 10^−5^	0.005219396	27	332	0.026286619
Pyruvate metabolism and Citric Acid (TCA) cycle	7	55	0.004816112	5.38 × 10^−5^	0.005219396	9	36	0.002850356
Smooth Muscle Contraction	6	39	0.003415061	6.65 × 10^−5^	0.005915444	5	11	8.71 × 10^−4^
Formation of tubulin folding intermediates by CCT/TriC	5	26	0.002276708	9.76 × 10^−5^	0.008006187	2	2	1.58 × 10^−4^
Prefoldin mediated transfer of substrate to CCT/TriC	5	28	0.002451839	1.38 × 10^−4^	0.010455726	2	2	1.58 × 10^−4^
Metabolism	58	2142	0.187565674	1.63 × 10^−4^	0.011603817	89	2001	0.158432304
The citric acid (TCA) cycle and respiratory electron transport	11	176	0.015411559	2.66 × 10^−4^	0.017547815	11	64	0.0050673
Cooperation of Prefoldin and TriC/CCT in actin and tubulin folding	5	33	0.002889667	2.92 × 10^−4^	0.018103534	6	6	4.75 × 10^−4^
Interleukin-12 family signaling	6	56	0.004903678	4.58 × 10^−4^	0.027046947	12	114	0.009026128
Protein ubiquitination	7	80	0.007005254	5.18 × 10^−4^	0.029024722	30	32	0.00253365
Selective autophagy	7	82	0.007180385	5.99 × 10^−4^	0.030593862	23	48	0.003800475
Citric acid cycle (TCA cycle)	4	22	0.001926445	6.12 × 10^−4^	0.030593862	5	17	0.001346002
Folding of actin by CCT/TriC	3	10	8.76 × 10^−4^	7.31 × 10^−4^	0.035103663	2	2	1.58 × 10^−4^
RHO GTPases activate PKNs	6	63	0.005516637	8.42 × 10^−4^	0.038746044	12	20	0.001583531
TP53 Regulates Metabolic Genes	7	88	0.007705779	9.01 × 10^−4^	0.039649516	5	34	0.002692003
Aggrephagy	5	44	0.00385289	0.001057731	0.044424721	13	15	0.001187648

**Table 2:** List of the 25 most relevant Reactome pathways overrepresented in R GBM tumor zone, in *p*-value decreasing order.

Pathway Name	Entities Found	Entities Total	Entities Ratio	Entities *p*-Value	Entities FDR	Reactions Found	Reactions Total	Reactions Ratio
Cellular responses to external stimuli	26	579	0.051	9.22 × 10^−10^	8.19 × 10^−7^	50	255	0.020190024
Cellular responses to stress	25	565	0.049	2.76 × 10^−9^	1.23 × 10^−6^	47	224	0.01773555
Eukaryotic Translation Elongation	8	95	0.008	1.21 × 10^−5^	0.003568961	6	9	7.13 × 10^−4^
L13a-mediated translational silencing of Ceruloplasmin expression	8	112	0.01	3.85 × 10^−5^	0.006094382	2	3	2.38 × 10^−4^
GTP hydrolysis and joining of the 60S ribosomal subunit	8	113	0.01	4.10 × 10^−5^	0.006094382	3	3	2.38 × 10^−4^
HSP90 chaperone cycle for steroid hormone receptors (SHR)	6	57	0.005	4.57 × 10^−5^	0.006094382	7	12	9.50 × 10^−4^
Metabolism of RNA	20	675	0.059	5.23 × 10^−5^	0.006094382	40	187	0.014806017
Cap-dependent Translation Initiation	8	120	0.011	6.22 × 10^−5^	0.006094382	17	18	0.001425178
Eukaryotic Translation Initiation	8	120	0.011	6.22 × 10^−5^	0.006094382	19	21	0.001662708
Regulation of Complement cascade	8	135	0.012	1.39 × 10^−4^	0.011833692	7	42	0.003325416
Response of EIF2AK4 (GCN2) to amino acid deficiency	7	102	0.009	1.52 × 10^−4^	0.011833692	6	16	0.001266825
Signaling by the B Cell Receptor (BCR)	9	176	0.015	1.60 × 10^−4^	0.011833692	18	43	0.003404592
Complement cascade	8	146	0.013	2.36 × 10^−4^	0.016016904	16	71	0.005621536
COPI-independent Golgi-to-ER retrograde traffic	5	53	0.005	3.30 × 10^−4^	0.019025205	2	7	5.54 × 10^−4^
Nonsense-Mediated Decay (NMD)	7	117	0.01	3.46 × 10^−4^	0.019025205	6	6	4.75 × 10^−4^
Nonsense Mediated Decay (NMD) enhanced by the Exon Junction Complex (EJC)	7	117	0.01	3.46 × 10^−4^	0.019025205	5	5	3.96 × 10^−4^
Hemostasis	19	726	0.064	3.98 × 10^−4^	0.019131188	13	332	0.026286619
Advanced glycosylation endproduct receptor signaling	3	13	0.001	4.32 × 10^−4^	0.019131188	3	4	3.17 × 10^−4^
Apoptosis induced DNA fragmentation	3	13	0.001	4.32 × 10^−4^	0.019131188	5	12	9.50 × 10^−4^
Translation	11	294	0.026	4.42 × 10^−4^	0.019131188	34	99	0.00783848
Innate Immune System	26	1187	0.104	5.06 × 10^−4^	0.019131188	125	662	0.052414885
Peptide chain elongation	6	90	0.008	5.27 × 10^−4^	0.019131188	4	5	3.96 × 10^−4^
Translation initiation complex formation	5	59	0.005	5.35 × 10^−4^	0.019131188	2	2	1.58 × 10^−4^
Ribosomal scanning and start codon recognition	5	59	0.005	5.35 × 10^−4^	0.019131188	2	2	1.58 × 10^−4^
Activation of the mRNA upon binding of the cap-binding complex and eIFs, and subsequent binding to 43S	5	60	0.005	5.77 × 10^−4^	0.019131188	5	6	4.75 × 10^−4^

**Table 3:** Protein elements shared between CUSA CORE and CUSA A-.

Uniprot Accession	Protein Name
**P02656**	Apolipoprotein CIII
**P16949**	Stathmin
**O75347**	**Tubulin-specific Chaperone A**
**P23527**	**Histone H2B type 1-O**
**O14979**	**Heterogeneous nuclear ribonucleoprotein Dlike**
**P25713**	Metallothionein-3 *
**Q16799**	Reticulon-1
**P68363**	Tubulin -1B chain
**P68032**	Actin, α cardiac muscle 1 *
**P0DOY3**	Immunoglobulin Lambda Constant 3 *
**Q13526**	Peptidyl-prolyl cis-trans isomerase NIMA-interacting 1 *

The proteins in bold showed statistically significantly different mean levels between the two zones (*p*-value < 0.05). * proteins presenting statistically significant higher mean levels in CUSA A- compared to CUSA core.

**Table 4:** List of the six proteins with statistically higher mean levels in CUSA A- with respect to CUSA A+ (*p*-value < 0.05).

Uniprot Accession	Protein Name
P00367	Glutamate dehydrogenase1, mytochondrial
P05164	Myeloperoxidase
P22177	Proliferating cell nuclear antigen
P17600	Synapsin-1
P60880	Synaptosomal-associated protein 25
Q16143	Beta-synuclein

**Table 5:** Molecular pathways exclusively classified in CUSA A- and CUSA A+. The pathways in bold were found over-represented by Panther tool following the gene ontology analysis of 193 proteins grouped as “tumor zone”.

Pathways CUSA A- Exclusive	Pathways CUSA A+ Exclusive
TGFβ signaling pathway	Adenine and hypoxanthine salvage pathway
	**5-Hydroxytryptamine degredation**
	Vitamin B6 metabolism
	Toll receptor signaling pathway
	p53 pathway by glucose deprivation
	Heme biosynthesis
	Axon guidance mediated by Slit/Robo
	Gamma-aminobutyric acid synthesis
	Xanthine and guanine salvage pathway
	**Serine glycine biosynthesis**
	Pyridoxal-5-phosphate biosynthesis
	Axon guidance mediated by netrin
	Aminobutyrate degradation
	p53 pathway feedback loops 2
	Ras Pathway

**Table 6:** List of the molecular pathways exclusively classified in R GBM CUSA CORE, CUSA A+, and CUSA A-.

CUSA CORE Exclusive Pathway	CUSA A+ Exclusive Pathway	CUSA A- Exclusive Pathways
TGFβ signaling pathway	Mannose metabolism	Pyridoxal phosphate salvage pathway
		Insulin/IGF pathway-protein kinase B signaling cascade
		Interleukin signaling Pathway
		Gamma-aminobutyric acid synthesis
		Endothelin signaling pathway
		PDGF signaling pathway
		Aminobutyrate degradation

**Table 7:** Lists in comparison of the nine proteins exclusive of ND GBM CORE with regards to R GBM CORE (column A) and of the nine proteins exclusive of ND GBM CORE with regards to ND GBM CUSA A+ and CUSA A- (column B).

(A) Exclusive CORE Proteins (ND vs. R, Figure 12)	(B) Exclusive CORE Proteins (ND CORE vs ND A+ vs. ND A-) (from Table S1)
**Q9BVA1: Tubulin beta 2B chain**	**Q9BVA1: Tubulin beta 2B chain**
**P13987: CD59 glycoprotein**	**P13987: CD59 glycoprotein**
**P10451: Osteopontin**	**P10451:Osteopontin**
**P07951:Tropomyosin beta chain**	**P07951:Tropomyosin beta chain**
Q14315: Filamin-C	P10412:Histone H1.4
P04264: Keratin, type II cytoskeletal 1	Q05682:Caldesmon
P0DOY2: Immunoglobulin lambda constant 2	P16070:CD44 antigen
P13645: Keratin, type I cytoskeletal 10	Q92945:Far upstream element-binding protein 2
P59665: Neutrophil defensin 1	P02795:Metallothionein-2

The name and the Uniprot accession number of each characterized protein is reported. Proteins in bold are those shared between the two lists of proteins.

**Table 8:** Patient data and GBM tumor location and state specifications under study.

Patient	Age	Location of Tumor	State of GBM Newly Diagnosed (ND) Recurrent (R)
PP1	57	Left parieto-occipital	ND
PP2	74	Left parietal	ND
PP3	52	Left parietal	ND
PP4	50	Left temporal	R
PP5	56	Right parietal	R
PP6	40	Right frontal	R
PP7	75	Right parieto-occipital	ND

## Supplementary Materials

The following are available online at https://www.mdpi.com/2072-6694/13/1/30/s1, Table S1: Uniprot accession, name and zone of identification data of the 193 proteins characterizing the tumor zone; Table S2: exported excel data file of the Proteome Discoverer software proteins and peptides identification in CORE, A+ and A- zones of ND GBM pools, as resulting from the multireport data elaboration of the analytical replicate; Table S3: exported excel data file of the Proteome Discoverer software proteins and peptides identification in CORE, A+ and A- zones of R GBM pools, as resulting from the multireport data elaboration of the analytical replicate. Table S4: Venn diagram sample grouping data of the protein identified in ND (S3_A) and R S3_(B) GBM pools. Table S5: Venn diagram sample grouping data of the pathways classified in ND (S4_A) and R (S4_B) GBM zones by PANTHER tool.

## References

[1] Suk K. (2012). Proteomic Analysis of Glioma Chemoresistance. Curr. Neuropharmacol..

[2] Jayaram S., Gupta M.K., Polisetty R.V., Cho W.C., Sirdeshmukh R. (2014). Towards developing biomarkers for glioblastoma multiforme: A proteomics view. Expert Rev. Proteom..

[3] Song Y.-C., Lu G.-X., Zhang H.-W., Zhong X.-M., Cong X.-L., Xue S.-B., Kong R., Li D., Chang Z.-Y., Wang X.-F. (2017). Proteogenomic characterization and integrative analysis of glioblastoma multiforme. Oncotarget.

[4] Silantyev A.S., Falzone L., Libra M., Gurina O.I., Kardashova K.S., Nikolouzakis T.K., Nosyrev A.E., Sutton C.W., Mitsias P.D., Tsatsakis A. (2019). Current and Future Trends on Diagnosis and Prognosis of Glioblastoma: From Molecular Biology to Proteomics. Cells.

[5] Pirlog R., Susman S., Iuga C., Florian I.S. (2019). Proteomic Advances in Glial Tumors through Mass Spectrometry Approaches. Medicina.

[6] Simon T., Jackson E., Giamas G. (2020). Breaking through the glioblastoma micro-environment via extracellular vesicles. Oncogene.

[7] Yekula A., Yekula A., Muralidharan K., Kang K., Carter B.S., Balaj L. (2020). Extracellular Vesicles in Glioblastoma Tumor Microenvironment. Front. Immunol..

[8] Hallal S., Russell B.P., Wei H., Lee M.Y.T., Toon C.W., Sy J., Shivalingam B., Buckland M.E., Kaufman K.L. (2019). Extracellular Vesicles from Neurosurgical Aspirates Identifies Chaperonin Containing TCP1 Subunit 6A as a Potential Glioblastoma Biomarker with Prognostic Significance. Proteomics.

[9] Mallawaaratchy D.M., Hallal S., Russell B., Ly L., Ebrahimkhani S., Wei H., Christopherson R.I., Buckland M.E., Kaufman K.L. (2017). Comprehensive proteome profiling of glioblastoma-derived extracellular vesicles identifies markers for more aggressive disease. J. Neuro Oncol..

[10] Whitehead C.A., Kaye A.H., Drummond K.J., Widodo S.S., Mantamadiotis T., Vella L.J., Stylli S.S. (2020). Extracellular vesicles and their role in glioblastoma. Crit. Rev. Clin. Lab. Sci..

[11] Feldman L., Fuchshuber P., Jones D.B. (2012). The SAGES Manual on the Fundamental Use of Surgical Energy (FUSE).

[12] Day B.W., Stringer B.W., Wilson J., Jeffree R.L., Jamieson P.R., Ensbey K.S., Bruce Z.C., Inglis P., Allan S., Winter C. (2013). Glioma Surgical Aspirate: A Viable Source of Tumor Tissue for Experimental Research. Cancers.

[13] Louis D.N., Perry A., Reifenberger G., Von Deimling A., Figarella-Branger D., Cavenee W.K., Ohgaki H., Wiestler O.D., Kleihues P., Ellison D.W. (2016). The 2016 World Health Organization Classification of Tumors of the Central Nervous System: A summary. Acta Neuropathol..

[14] Monticelli M., Zeppa P., Zenga F., Altieri R., Mammi M., Bertero L., Castellano I., Cassoni P., Melcarne A., La Rocca G. (2018). The post-surgical era of GBM: How molecular biology has impacted on our clinical management. A review. Clin. Neurol. Neurosurg..

[15] Ius T., Pignotti F., Della Pepa G.M., Bagatto D., Isola M., Battistella C., Gaudino S., Pegolo E., Chiesa S., Arcicasa M. (2020). Glioblastoma: From volumetric analysis to molecular predictors. J. Neurosurg. Sci..

[16] Kim H., Park Y.J. (2018). Links between Serine Biosynthesis Pathway and Epigenetics in Cancer Metabolism. Clin. Nutr. Res..

[17] Kathagen-Buhmann A., Schulte A., Weller J., Holz M., Herold-Mende C., Glass R., Lamszus K. (2016). Glycolysis and the pentose phosphate pathway are differentially associated with the dichotomous regulation of glioblastoma cell migration versus proliferation. Neuro Oncol..

[18] Wang X., Yang K., Xie Q., Wu Q., Mack S.C., Shi Y., Kim L.J.Y., Prager B.C., Flavahan W.A., Liu X. (2017). Purine synthesis promotes maintenance of brain tumor initiating cells in glioma. Nat. Neurosci..

[19] Merzak A., Koochekpour S., Fillion M.-P., Fillion G., Pilkington G.J. (1996). Expression of serotonin receptors in human fetal astrocytes and glioma cell lines: A possible role in glioma cell proliferation and migration. Mol. Brain Res..

[20] Vasilev A., Sofi R., Tong L., Teschemacher A.G., Kasparov S. (2018). In Search of a Breakthrough Therapy for Glioblastoma Multiforme. Neuroglia.

[21] Plun-Favreau H., Lewis P.A., Hardy J., Martins L.M., Wood N. (2010). Cancer and Neurodegeneration: Between the Devil and the Deep Blue Sea. PLoS Genet..

[22] Mooney K.L., Choy W., Sidhu S., Pelargos P., Bui T.T., Voth B., Barnette N., Yang I. (2016). The role of CD44 in glioblastoma multiforme. J. Clin. Neurosci..

[23] Stupp R., Hegi M.E., Mason W.P., Bent M.J.V.D., Taphoorn M.J.B., Janzer R.C., Ludwin S.K., Allgeier A., Fisher B., Belanger K. (2009). Effects of radiotherapy with concomitant and adjuvant temozolomide versus radiotherapy alone on survival in glioblastoma in a randomised phase III study: 5-year analysis of the EORTC-NCIC trial. Lancet Oncol..

[24] Pietras A., Katz A.M., Ekström E.J., Wee B., Halliday J.J., Pitter K.L., Werbeck J.L., Amankulor N.M., Huse J.T., Holland E.C. (2014). Osteopontin-CD44 Signaling in the Glioma Perivascular Niche Enhances Cancer Stem Cell Phenotypes and Promotes Aggressive Tumor Growth. Cell Stem Cell.

[25] Vaillant B.D., Bhat K., Sulman E.P., Balasubramaniyan V., Wang S., Aldape K.D., Colman H. (2011). CD44 as a prognostic and predictive marker for GBM. J. Clin. Oncol..

[26] Lim S., Kim D., Ju S., Shin S., Cho I.-J., Park S., Grailhe R., Lee C., Kim Y.K. (2018). Glioblastoma-secreted soluble CD44 activates tau pathology in the brain. Exp. Mol. Med..

[27] Wei J., Marisetty A., Schrand B., Gabrusiewicz K., Hashimoto Y., Ott M., Grami Z., Kong L.-Y., Ling X., Caruso H.G. (2018). Osteopontin mediates glioblastoma-associated macrophage infiltration and is a potential therapeutic target. J. Clin. Investig..

[28] D’Alessio A., Proietti G., Sica G., Scicchitano B.M. (2019). Pathological and Molecular Features of Glioblastoma and Its Peritumoral Tissue. Cancers.

[29] Zhang R., Liu Q., Liao Q., Zhao Y. (2018). CD59: A promising target for tumor immunotherapy. Futur. Oncol..

[30] Zheng P.-P., Sieuwerts A.M., Luider T.M., Van Der Weiden M., Sillevis-Smitt P.A., Kros J.M. (2004). Differential Expression of Splicing Variants of the Human Caldesmon Gene (CALD1) in Glioma Neovascularization versus Normal Brain Microvasculature. Am. J. Pathol..

[31] Uhlén M., Zhang C., Lee S., Sjöstedt E., Fagerberg L., Bidkhori G., Benfeitas R., Arif M., Liu Z., Edfors F. (2017). A pathology atlas of the human cancer transcriptome. Science.

[32] Pontén F., Jirström K., Uhlen M. (2008). The Human Protein Atlas—A tool for pathology. J. Pathol..

[33] Data Available from v19.proteinatlas.org. https://www.proteinatlas.org/ENSG00000137285-TUBB2B/pathology.

[34] Masiulionytė B., Valiulyte I., Tamašauskas A., Skiriutė D. (2019). Metallothionein Genes are Highly Expressed in Malignant Astrocytomas and Associated with Patient Survival. Sci. Rep..

[35] Si M., Lang J. (2018). The roles of metallothioneins in carcinogenesis. J. Hematol. Oncol..

[36] Rupp T., Langlois B., Koczorowska M.M., Radwanska A., Sun Z., Hussenet T., Lefebvre O., Murdamoothoo D., Arnold C., Klein A. (2016). Tenascin-C Orchestrates Glioblastoma Angiogenesis by Modulation of Pro- and Anti-angiogenic Signaling. Cell Rep..

[37] Morales F.C., Molina J.R., Hayashi Y., Georgescu M.-M. (2010). Overexpression of ezrin inactivates NF2 tumor suppressor in glioblastoma. Neuro Oncol..

[38] Kotb A., Hyndman M.E., Patel T.R. (2018). The role of zyxin in regulation of malignancies. Heliyon.

[39] Wen X.-M., Luo T., Jiang Y., Wang L.-H., Luo Y., Chen Q., Yang K., Yuan Y., Luo C., Zhang X. (2020). Zyxin (ZYX) promotes invasion and acts as a biomarker for aggressive phenotypes of human glioblastoma multiforme. Lab. Investig..

[40] Data Available from v19.proteinatlas.org. https://www.proteinatlas.org/ENSG00000152795-HNRNPDL/pathology#gene_information.

[41] Data Available from v19.proteinatlas.org. https://www.proteinatlas.org/search/protein_class:Cancer-related+genes.

[42] Data Available from v19.proteinatlas.org. https://www.proteinatlas.org/ENSG00000117632-STMN1/pathology.

[43] Data Available from v19.proteinatlas.org. https://www.proteinatlas.org/ENSG00000148672-GLUD1/pathology.

[44] Kutwin M., Sawosz E., Jaworski S., Wierzbicki M., Strojny B., Grodzik M., Chwalibog A. (2017). Assessment of the proliferation status of glioblastoma cell and tumour tissue after nanoplatinum treatment. PLoS ONE.

[45] Data Available from v19.proteinatlas.org. https://www.proteinatlas.org/ENSG00000008056-SYN1/pathology#gene_information.

[46] Cesca F., Baldelli P., Valtorta F., Benfenati F. (2010). The synapsins: Key actors of synapse function and plasticity. Prog. Neurobiol..

[47] Conti F. (2010). Fisiologia Medica 1.

[48] Tanji K., Mori F., Nakajo S., Imaizumi T., Yoshida H., Hirabayashi T., Yoshimoto M., Satoh K., Takahashi H., Wakabayashi K. (2001). Expression of β-synuclein in normal human astrocytes. NeuroReport.

[49] Data Available from v19.proteinatlas.org. https://www.proteinatlas.org/ENSG00000132639-SNAP25/pathology.

[50] Strzalka W., Ziemienowicz A. (2010). Proliferating cell nuclear antigen (PCNA): A key factor in DNA replication and cell cycle regulation. Ann. Bot..

[51] Maga G. (2003). Proliferating cell nuclear antigen (PCNA): A dancer with many partners. J. Cell Sci..

[52] Data Available from v19.proteinatlas.org. https://www.proteinatlas.org/ENSG00000178802-MPI/pathology.

[53] Mi H., Huang X., Muruganujan A., Tang H., Mills C., Kang D., Thomas P.D. (2017). PANTHER version 11: Expanded annotation data from Gene Ontology and Reactome pathways, and data analysis tool enhancements. Nucleic Acids Res..

[54] Accession P00019. http://www.pantherdb.org/pathway/pathDetail.do?clsAccession=P00019.

[55] Accession P00047. http://www.pantherdb.org/pathway/pathDetail.do?clsAccession=P00047.

[56] Popescu A.M., Alexandru O., Brindusa C., Purcaru S.O., Tache D.E., Tataranu L.G., Taisescu C., Dricu A. (2015). Targeting the VEGF and PDGF signaling pathway in glioblastoma treatment. Int. J. Clin. Exp. Pathol..

[57] Della Pepa G.M., Menna G., Ius T., Di Bonaventura R., Altieri R., Marchese E., Olivi A., Sabatino G., La Rocca G. (2020). Contrast enhanced ultrasound (CEUS) applications in neurosurgical and neurological settings—New scenarios for brain and spinal cord ultrasonography. A systematic review. Clin. Neurol. Neurosurg..

[58] Altieri R., Melcarne A., Di Perna G., Specchia F.M.C., Fronda C., La Rocca G., Cofano F., Sabatino G., Della Pepa G.M., Olivi A. (2018). Intra-Operative Ultrasound: Tips and Tricks for Making the Most in Neurosurgery. Surg. Technol. Int..

[59] Della Pepa G.M., Ius T., La Rocca G., Gaudino S., Isola M., Pignotti F., Rapisarda A., Mazzucchi E., Giordano C., Dragonetti V. (2020). 5-Aminolevulinic Acid and Contrast-Enhanced Ultrasound: The Combination of the Two Techniques to Optimize the Extent of Resection in Glioblastoma Surgery. Neurosurgery.

[60] Della Pepa G.M., Sabatino G., La Rocca G. (2019). “Enhancing Vision” in High Grade Glioma Surgery: A Feasible Integrated 5-ALA + CEUS Protocol to Improve Radicality. World Neurosurg..

[61] La Rocca G., Della Pepa G.M., Menna G., Altieri R., Ius T., Rapisarda A., Olivi A., Sabatino G. (2019). State of the art of fluorescence guided techniques in neurosurgery. J. Neurosurg. Sci..

[62] Manini I., Caponnetto F., Dalla E., Ius T., Della Pepa G.M., Pegolo E., Bartolini A., La Rocca G., Menna G., Di Loreto C. (2020). Heterogeneity Matters: Different Regions of Glioblastoma Are Characterized by Distinctive Tumor-Supporting Pathways. Cancers.

[63] Deutsch E.W., Overall C.M., van Eyk J.E., Baker M.S., Paik Y.-K., Weintraub S.T., Lane L., Martens L., Vandenbrouck Y., Kusebauch U. (2016). Human Proteome Project Mass Spectrometry Data Interpretation Guidelines 2.1. J. Proteome Res..

[64] Fabregat A., Sidiropoulos K., Viteri G., Forner-Martinez O., Marin-Garcia P., Arnau V., D’Eustachio P., Stein L., Hermjakob H. (2017). Reactome pathway analysis: A high-performance in-memory approach. BMC Bioinform..

[65] Szklarczyk D., Gable A.L., Lyon D., Junge A., Wyder S., Huerta-Cepas J., Simonovic M., Doncheva N.T., Morris J.H., Bork P. (2019). STRING v11: Protein–Protein association networks with increased coverage, supporting functional discovery in genome-wide experimental datasets. Nucleic Acids Res..

