# Supplementary material for: Glioblastoma CUSA Fluid Protein Profiling: A Comparative Investigation of the Core and Peripheral Tumor Zones
Source: Cancers (Basel). 2020 Dec 23;13(1):30. doi: 10.3390/cancers13010030 (PMC7795841; doi:10.3390/cancers13010030)
Supplement: Supplementary file 1 [file cancers-13-00030-s001.zip › cancers-992405_supplementary_proof/cancers-992405_supplementary_conversion.docx]

**Table S1.** Uniprot accession, name and zone of identification data of the 193 proteins characterizing the tumor zone.

| **Uniprot Accession** | **Protein Name** | **Zone of identification** |
| --- | --- | --- |
| Q9BVA1 | Tubulin 2B chain | CUSA CORE |
| P13987 | CD59 glycoprotein | CUSA CORE |
| Q92945 | Far upstream element-binding protein 2 | CUSA CORE |
| P16070 | CD44 antigen | CUSA CORE |
| P10412 | Histone H1.4 | CUSA CORE |
| Q05682 | Caldesmon | CUSA CORE |
| P10451 | Osteopontin | CUSA CORE |
| P07951 | Tropomyosin chain | CUSA CORE |
| P02795 | Metallothionein-2 | CUSA CORE |
| P04264 | Keratin, type II cytoskeletal 1 | CUSA CORE and CUSA A+ |
| P15311 | Ezrin* | CUSA CORE and CUSA A+ |
| P24821 | Tenascin | CUSA CORE and CUSA A+ |
| P02750 | Leucine-rich alpha-2-glycoprotein* | CUSA CORE and CUSA A+ |
| P13645 | Keratin, type I cytoskeletal 10 | CUSA CORE and CUSA A+ |
| P0DOY2 | Immunoglobulin lambda constant 2* | CUSA CORE and CUSA A+ |
| P05386 | 60S acidic ribosomal protein P1* | CUSA CORE and CUSA A+ |
| Q15942 | Zyxin | CUSA CORE and CUSA A+ |
| P09211 | Glutathione S-transferase P | CUSA A+ |
| P08758 | Annexin A5 | CUSA A+ |
| O60814 | Histone H2B type 1-K | CUSA A+ |
| P60201 | Myelin proteolipid protein | CUSA A+ |
| P00736 | Complement C1r subcomponent | CUSA A+ |
| P05155 | Plasma protease C1 inhibitor | CUSA A+ |
| P12532 | Creatine kinase U-type, mitochondrial | CUSA A+ |
| P13639 | Elongation factor 2 | CUSA A+ |
| P13489 | Ribonuclease inhibitor | CUSA A+ |
| Q86VP6 | Cullin-associated NEDD8-dissociated protein 1 | CUSA A+ |
| P01019 | Angiotensinogen | CUSA A+ |
| Q9BRA2 | Thioredoxin domain-containing protein 17 | CUSA A+ |
| Q15836 | Vesicle-associated membrane protein 3 | CUSA A+ |
| P00748 | Coagulation factor XII | CUSA A+ |
| Q9Y6R7 | IgGFc-binding protein | CUSA A+ |
| Q9NTK5 | Obg-like ATPase 1 | CUSA A+ |
| O95336 | 6-phosphogluconolactonase | CUSA A+ |
| Q13765 | Nascent polypeptide-associated complex subunit alpha | CUSA A+ |
| Q9UN36 | Protein NDRG2 | CUSA A+ |
| P07203 | Glutathione peroxidase 1 | CUSA A+ |
| Q96KP4 | Cytosolic non-specific dipeptidase | CUSA A+ |
| P28066 | Proteasome subunit alpha type-5 | CUSA A+ |
| P31930 | Cytochrome b-c1 complex subunit 1, mitochondrial | CUSA A+ |
| P31939 | Bifunctional purine biosynthesis protein PURH | CUSA A+ |
| Q15056 | Eukaryotic translation initiation factor 4H (eIF-4H) | CUSA A+ |
| P78371 | T-complex protein 1 subunit beta (TCP-1-beta) | CUSA A+ |
| O00244 | Copper transport protein ATOX1 | CUSA A+ |
| Q15365 | Poly(rC)-binding protein 1 | CUSA A+ |
| P47755 | F-actin-capping protein subunit alpha-2 | CUSA A+ |
| P11216 | Glycogen phosphorylase, brain form | CUSA A+ |
| P48735 | Isocitrate dehydrogenase [NADP], mitochondrial | CUSA A+ |
| P04179 | Superoxide dismutase [Mn], mitochondrial | CUSA A+ |
| P23297 | Protein S100-A1 | CUSA A+ |
| P61158 | Actin-related protein 3 | CUSA A+ |
| P60953 | Cell division control protein 42 homolog | CUSA A+ |
| O43707 | Alpha-actinin-4 | CUSA A+ |
| P62258 | 14-3-3 protein epsilon | CUSA A+ |
| P37837 | Transaldolase | CUSA A+ |
| P07339 | Cathepsin D | CUSA A+ |
| Q32P51 | Heterogeneous nuclear ribonucleoprotein A1-like 2 (hnRNP A1-like 2) | CUSA A+ |
| P17655 | Calpain-2 catalytic subunit | CUSA A+ |
| P06744 | Glucose-6-phosphate isomerase (GPI) | CUSA A+ |
| P14625 | Endoplasmin | CUSA A+ |
| Q14194 | Dihydropyrimidinase-related protein 1 (DRP-1) | CUSA A+ |
| Q99832 | T-complex protein 1 subunit eta (TCP-1-eta) | CUSA A+ |
| P22314 | Ubiquitin-like modifier-activating enzyme 1 | CUSA A+ |
| Q92928 | Putative Ras-related protein Rab-1C | CUSA A+ |
| O75390 | Citrate synthase, mitochondrial | CUSA A+ |
| P36222 | Chitinase-3-like protein 1 | CUSA A+ |
| Q96E39 | RNA binding motif protein, X-linked-like-1 | CUSA A+ |
| Q99536 | Synaptic vesicle membrane protein VAT-1 homolog | CUSA A+ |
| Q92752 | Tenascin-R | CUSA A+ |
| P61266 | Syntaxin-1B | CUSA A+ |
| P99999 | Cytochrome c | CUSA A+ |
| P04083 | Annexin A1 | CUSA A+ |
| P22234 | Multifunctional protein ADE2 | CUSA A+ |
| O00299 | Chloride intracellular channel protein 1 | CUSA A+ |
| P36955 | Pigment epithelium-derived factor (PEDF) | CUSA A+ |
| Q01518 | Adenylyl cyclase-associated protein 1 (CAP 1) | CUSA A+ |
| P30153 | Serine/threonine-protein phosphatase 2A 65 kDa regulatory subunit A alpha isoform | CUSA A+ |
| P17931 | Galectin-3 (Gal-3) | CUSA A+ |
| P67775 | Serine/threonine-protein phosphatase 2A catalytic subunit alpha isoform | CUSA A+ |
| P48681 | Nestin | CUSA A+ |
| P10768 | S-formylglutathione hydrolase | CUSA A+ |
| Q9UJ70 | N-acetyl-D-glucosamine kinase | CUSA A+ |
| P13073 | Cytochrome c oxidase subunit 4 isoform 1, mitochondrial | CUSA A+ |
| P68036 | Ubiquitin-conjugating enzyme E2 L3 | CUSA A+ |
| Q01105 | Protein SET | CUSA A+ |
| Q08380 | Galectin-3-binding protein | CUSA A+ |
| P21796 | Voltage-dependent anion-selective channel protein 1 | CUSA A+ |
| P27824 | Calnexin | CUSA A+ |
| P61626 | Lysozyme C | CUSA A+ |
| P54727 | UV excision repair protein RAD23 homolog B | CUSA A+ |
| P22695 | Cytochrome b-c1 complex subunit 2, mitochondrial | CUSA A+ |
| P00505 | Aspartate aminotransferase, mitochondrial | CUSA A+ |
| Q04917 | 14-3-3 protein eta | CUSA A+ |
| P07954 | Fumarate hydratase, mitochondrial | CUSA A+ |
| P05455 | Lupus La protein | CUSA A+ |
| Q9Y617 | Phosphoserine aminotransferase | CUSA A+ |
| P27169 | Serum paraoxonase/arylesterase 1 | CUSA A+ |
| P00492 | Hypoxanthine-guanine phosphoribosyltransferase | CUSA A+ |
| P09871 | Complement C1s subcomponent | CUSA A+ |
| P53396 | ATP-citrate synthase | CUSA A+ |
| P61981 | 14-3-3 protein gamma | CUSA A+ |
| P23381 | Tryptophan--tRNA ligase, cytoplasmic | CUSA A+ |
| P00533 | Epidermal growth factor receptor | CUSA A+ |
| P21266 | Glutathione S-transferase Mu 3 | CUSA A+ |
| P61088 | Ubiquitin-conjugating enzyme E2 N | CUSA A+ |
| P54725 | UV excision repair protein RAD23 homolog A | CUSA A+ |
| Q00610 | Clathrin heavy chain 1 | CUSA A+ |
| P49418 | Amphiphysin | CUSA A+ |
| P30626 | Sorcin | CUSA A+ |
| P43004 | Excitatory amino acid transporter 2 | CUSA A+ |
| P30044 | Peroxiredoxin-5, mitochondrial | CUSA A+ |
| Q8N111 | Cell cycle exit and neuronal differentiation protein 1 | CUSA A+ |
| P02747 | Complement C1q subcomponent subunit C | CUSA A+ |
| P01619 | Immunoglobulin kappa variable 3-20 | CUSA A+ |
| P28838 | Cytosol aminopeptidase | CUSA A+ |
| O43175 | D-3-phosphoglycerate dehydrogenase | CUSA A+ |
| P36871 | Phosphoglucomutase-1 (PGM 1) | CUSA A+ |
| P20073 | Annexin A7 | CUSA A+ |
| P02743 | Serum amyloid P-component (SAP) | CUSA A+ |
| P80404 | 4-aminobutyrate aminotransferase, mitochondrial | CUSA A+ |
| P50897 | Palmitoyl-protein thioesterase 1 (PPT-1) | CUSA A+ |
| P38606 | V-type proton ATPase catalytic subunit A | CUSA A+ |
| P01700 | Immunoglobulin lambda variable 1-47 | CUSA A+ |
| P37802 | Transgelin-2 | CUSA A+ |
| Q96PD5 | N-acetylmuramoyl-L-alanine amidase Uniprot | CUSA A+ |
| O75083 | WD repeat-containing protein 1 | CUSA A+ |
| Q13642 | Four and a half LIM domains protein 1 (FHL-1) | CUSA A+ |
| P02654 | Apolipoprotein C-I | CUSA A+ |
| Q9H4G4 | Golgi-associated plant pathogenesis-related protein 1 (GAPR-1) | CUSA A+ |
| P00338 | L-lactate dehydrogenase A chain (LDH-A) | CUSA A+ |
| Q15366 | Poly(rC)-binding protein 2 | CUSA A+ |
| Q15181 | Inorganic pyrophosphatase | CUSA A+ |
| P45974 | Ubiquitin carboxyl-terminal hydrolase 5 | CUSA A+ |
| P31943 | Heterogeneous nuclear ribonucleoprotein H (hnRNP H) | CUSA A+ |
| P16401 | Histone H1.5 | CUSA A+ |
| P62805 | Histone H4 | CUSA A+ |
| P07355 | Annexin A2 | CUSA A+ |
| P07602 | Prosaposin | CUSA A+ |
| P04080 | Cystatin-B | CUSA A+ |
| P13667 | Protein disulfide-isomerase A4 | CUSA A+ |
| P08697 | Alpha-2-antiplasmin | CUSA A+ |
| Q92597 | Protein NDRG1 | CUSA A+ |
| P34932 | Heat shock 70 kDa protein 4 | CUSA A+ |
| P18206 | Vinculin | CUSA A+ |
| P13716 | Delta-aminolevulinic acid dehydratase | CUSA A+ |
| P52209 | 6-phosphogluconate dehydrogenase, decarboxylating | CUSA A+ |
| P52565 | Rho GDP-dissociation inhibitor 1 (Rho GDI 1) | CUSA A+ |
| P0CG48 | Polyubiquitin-C | CUSA A+ |
| P21281 | V-type proton ATPase subunit B, brain isoform | CUSA A+ |
| Q9Y696 | Chloride intracellular channel protein 4 | CUSA A+ |
| P08133 | Annexin A6 | CUSA A+ |
| P26447 | Protein S100-A4 | CUSA A+ |
| P07237 | Protein disulfide-isomerase (PDI) | CUSA A+ |
| Q04760 | Lactoylglutathione lyase | CUSA A+ |
| P08865 | 40S ribosomal protein SA | CUSA A+ |
| P50213 | Isocitrate dehydrogenase [NAD] subunit alpha, mitochondrial | CUSA A+ |
| P31949 | Protein S100-A11 | CUSA A+ |
| P02753 | Retinol-binding protein 4 | CUSA A+ |
| P69892 | Hemoglobin subunit gamma-2 | CUSA A+ |
| P61764 | Syntaxin-binding protein 1 | CUSA A+ |
| P12814 | Alpha-actinin-1 | CUSA A+ |
| P49189 | 4-trimethylaminobutyraldehyde dehydrogenase | CUSA A+ |
| P21926 | CD9 antigen | CUSA A+ |
| Q14847 | LIM and SH3 domain protein 1 (LASP-1) | CUSA A+ |
| P27797 | Calreticulin | CUSA A+ |
| P16152 | Carbonyl reductase | CUSA A+ |
| P23526 | Adenosylhomocysteinase | CUSA A+ |
| P00352 | Retinal dehydrogenase 1 (RALDH 1) | CUSA A+ |
| Q14204 | Cytoplasmic dynein 1 heavy chain 1 | CUSA A+ |
| Q9UQM7 | Calcium/calmodulin-dependent protein kinase type II subunit alpha | CUSA A+ |
| P07108 | Acyl-CoA-binding protein (ACBP) | CUSA A+ |
| Q15084 | Protein disulfide-isomerase A6 | CUSA A+ |
| Q14974 | Importin subunit beta-1 | CUSA A+ |
| P30050 | 60S ribosomal protein L12 | CUSA A+ |
| P30046 | D-dopachrome decarboxylase | CUSA A+ |
| P43490 | Nicotinamide phosphoribosyltransferase | CUSA A+ |
| P41222 | Prostaglandin-H2 D-isomerase | CUSA A+ |
| P55795 | Heterogeneous nuclear ribonucleoprotein H2 (hnRNP H2) | CUSA A+ |
| P04439 | HLA class I histocompatibility antigen, A alpha chain | CUSA A+ |
| P68366 | Tubulin alpha-4A chain | CUSA A+ |
| P61204 | ADP-ribosylation factor 3 | CUSA A+ |
| P15531 | Nucleoside diphosphate kinase A | CUSA A+ |
| Q562R1 | Beta-actin-like protein 2 | CUSA A+ |
| P49368 | T-complex protein 1 subunit gamma | CUSA A+ |
| P35527 | Keratin, type I cytoskeletal 9 (Cytokeratin-9) (CK-9) (Keratin-9) (K9) | CUSA A+ |
| Q15417 | Calponin-3 | CUSA A+ |
| P49419 | Alpha-aminoadipic semialdehyde dehydrogenase | CUSA A+ |
| P50395 | Rab GDP dissociation inhibitor beta | CUSA A+ |
| P35637 | RNA-binding protein FUS | CUSA A+ |
| P63241 | Eukaryotic translation initiation factor 5A-1 | CUSA A+ |
| O60664 | Perilipin-3 | CUSA A+ |
| P13010 | X-ray repair cross-complementing protein 5 | CUSA A+ |
| P61586 | Transforming protein RhoA | CUSA A+ |
